# Glucagon-like peptide 1 agonists for treatment of patients with type 2 diabetes who fail metformin monotherapy: systematic review and meta-analysis of economic evaluation studies

**DOI:** 10.1136/bmjdrc-2019-001020

**Published:** 2020-07-19

**Authors:** Bhavani Shankara Bagepally, Usa Chaikledkaew, Yogesh Krishnarao Gurav, Thunyarat Anothaisintawee, Sitaporn Youngkong, Nathorn Chaiyakunapruk, Mark McEvoy, John Attia, Ammarin Thakkinstian

**Affiliations:** 1 Non-Communicable Diseases, ICMR-National Institute of Epidemiology, Chennai, India; 2 Mahidol University Health Technology Assessment (MUHTA) Graduate Program, Mahidol University, Bangkok, Thailand; 3 Social and Administrative Pharmacy Division, Department of Pharmacy, Faculty of Pharmacy, Mahidol University, Bangkok, Thailand; 4 Epidemiology Group, ICMR-National Institute of Virology, Pune, India; 5 Department of Family Medicine, Faculty of Medicine Ramathibodi Hospital, Mahidol University, Bangkok, Thailand; 6 College of Pharmacy, University of Utah, Salt Lake City, Utah, USA; 7 Centre for Clinical Epidemiology and Biostatistics, Hunter Medical Research Institute, School of Medicine and Public Health, University of Newcastle, New Lambton, New South Wales, Australia; 8 Division of Medicine, John Hunter Hospital, New Lambton, New South Wales, Australia; 9 Department of Clinical Epidemiology and Biostatistics, Faculty of Medicine Ramathibodi Hospital, Mahidol University, Bangkok, Thailand

**Keywords:** Glucagon-Like Peptide-1 (GLP-1), economic analysis, cost-effectiveness, meta-analysis

## Abstract

**Objectives:**

To conduct a systematic review and meta-analysis and to pool the incremental net benefits (INBs) of glucagon-like peptide 1 (GLP1) compared with other therapies in type 2 diabetes mellitus (T2DM) after metformin monotherapy failure.

**Research design and methods:**

The study design is a systematic review and meta-analysis. We searched MEDLINE (via PubMed), Scopus and Tufts Registry for eligible cost–utility studies up to June 2018, adhering to the Preferred Reporting Items for Systematic Reviews and Meta-Analyses guideline. We conducted a systematic review and pooled the INBs of GLP1s compared with other therapies in T2DM after metformin monotherapy failure. Various monetary units were converted to purchasing power parity, adjusted to 2017 US$. The INBs were calculated and then pooled across studies, stratified by level of country income; a random-effects model was used if heterogeneity was present, and a fixed-effects model if it was absent. Heterogeneity was assessed using Q test and I^2^ statistic.

**Results:**

A total of 56 studies were eligible, mainly from high-income countries (HICs). The pooled INBs of GLP1s compared with dipeptidyl peptidase-4 inhibitor (DPP4i) (n=10), sulfonylureas (n=6), thiazolidinedione (TZD) (n=3), and insulin (n=23) from HICs were US$4012.21 (95% CI US$−571.43 to US$8595.84, I^2^=0%), US$3857.34 (95% CI US$−7293.93 to US$15 008.61, I^2^=45.9%), US$37 577.74 (95% CI US$−649.02 to US$75 804.50, I^2^=92.4%) and US$14 062.42 (95% CI US$8168.69 to US$19 956.15, I^2^=86.4%), respectively. GLP1s were statistically significantly cost-effective compared with insulins, but not compared with DPP4i, sulfonylureas, and TZDs. Among GLP1s, liraglutide was more cost-effective compared with lixisenatide, but not compared with exenatide, with corresponding pooled INBs of US$4555.09 (95% CI US$3992.60 to US$5117.59, I^2^=0) and US$728.46 (95% CI US$−1436.14 to US$2893.07, I^2^=0), respectively.

**Conclusion:**

GLP1 agonists are a cost-effective choice compared with insulins, but not compared with DPP4i, sulfonylureas and TZDs.

**PROSPERO registration number:**

CRD42018105193.

**Significance of this study**

**What is already known about this subject?**

- Glucagon-like peptide 1 (GLP1) agonists are clinically effective in treating patients with type 2 diabetes mellitus (T2DM) who fail metformin monotherapy.
- Several economic evaluation studies, along with systematic reviews of the cost-effectiveness of GLP1 agonists, have already been conducted, but these have only been descriptive and results have been conflicting.

**What are the new findings?**

- We synthesized quantitative evidence of the cost-effectiveness of GLP1 agonists using a novel methodological approach.
- GLP1 agonists were significantly cost-effective compared with insulins but were not as cost-effective as dipeptidyl peptidase-4 inhibitor (DPP4i), sulfonylureas and thiazolidines in high income countries.
- Among GLP1 agonists, liraglutide was more cost-effective compared with lixisenatide, but not compared with exenatide.

**How might these results change the focus of research or clinical practice?**

- This study provides a novel approach to conducting a meta-analysis of economic evaluation studies.
- GLP1 drugs could be a better choice, compared with insulins, in treating patients with T2DM after metformin monotherapy failure, but not compared with other second-line drugs that is, DPP4i, sulfonylureas and thiazolidines.

## Introduction

Type 2 diabetes mellitus (T2DM) accounts for ~12% of the global health expenditure^1^ or US $850 billion per year.^1^ Its complications place a large social and financial burden on patients, families, and healthcare systems globally,^2^ leading to marked morbidity and mortality worldwide.^2^ Therefore, T2DM treatments aim to reduce long-term complications and mortality through glycaemic control.^3 4^ Pharmacotherapy guidelines suggest that metformin should be used as initial monotherapy and that second-line agents should be added if metformin monotherapy fails to maintain glycaemic control. Second-line agents include sulfonylurea, thiazolidinedione (TZD), dipeptidyl peptidase-4 inhibitor (DPP4i), sodium glucose co-transporter 2 (SGLT2) inhibitor, glucagon-like peptide 1 (GLP1) receptor agonist, and insulin.^5^

Recent network meta-analyses^6 7^ found that GLP1 agonists were not significantly different in terms of glycaemic control, mortality or safety compared with other antidiabetic drugs. However, the American Association of Clinical Endocrinologists^8^ suggests that GLP1s (ie, exenatide, liraglutide, dulaglutide, lixisenatide, albiglutide and semaglutide) should be preferentially used as second-line drugs added to metformin in patients who fail metformin monotherapy.

Current reviews of economic studies have been conducted but have some limitations, including providing only descriptive summaries^9–15^ and conducting no systematic searches.^9 12 15^ In addition, they considered only cost of illness, burden of illness, or clinical effectiveness,^10 12–14^ but not cost-effectiveness. None of these studies synthesized or pooled economic outcome measures, which would indicate overall cost-effectiveness of the interventions. Such information would be of immense help for decision making in countries where there is a lack of cost-effectiveness evidence or where there is limited capacity to generate such evidence.

Meta-analysis of economic evaluation studies was first developed by Crespo *et al*,^16^ and our research teams have recently further developed their methods to cover all aspects of meta-analysis.^9 10^ The incremental net benefit (INB) is calculated by multiplying willingness to pay (WTP) (per unit) and change in effectiveness (in units) and then subtracting the difference in costs; this estimate of INB is then pooled across studies, taking into account within-study and between-study variations. However, individual studies are conducted in different countries where WTP is different. Therefore, pooling INBs should be performed while stratifying by level of country income according to the World Bank classification.^17 18^ Positive and negative INBs refer to cost-effectiveness and non-cost-effectiveness, respectively.

Therefore, this systematic review and meta-analysis was conducted to pool the INBs of GLP1s compared with other antidiabetic treatments in patients who failed metformin monotherapy, stratified by the level of country income.

## Methods

We followed the Preferred Reporting Items for Systematic Reviews and Meta-Analysis Protocols,^11^ and the protocol was registered at PROSPERO (CRD42018105193).

### Data sources and search strategy

Searching was performed in PubMed, Scopus, the Cochrane Central Register of Controlled Trials, Wiley Library, ProQuest, and the Cost-effective Analysis Registry^12^ by Tufts Medical Center, from inception to June 2018 (see online supplementary appendix I). Economic evaluation studies were eligible if they met all the following inclusion criteria: patients with T2DM who failed metformin monotherapy, comparison of GLP1s with any second-line drug, and report of any economic outcomes, including incremental cost-effectiveness ratio (ICER), quality-adjusted life years (QALY), or INB. Studies related to clinical effectiveness, cost/burden of illness, reviews, or multiple publications were excluded.

10.1136/bmjdrc-2019-001020.supp1 Supplementary data

### Data extraction and risk of bias assessment

An a priori data extraction form was constructed consisting of study/patient characteristics, intervention, economic outcomes/parameters, the methods used, and data for pooling. For the economic parameters, cost, C, or incremental/delta costs, ΔC; clinical effectiveness, E, or its incremental/delta, ΔE; ICERs; measures of dispersion (ie, SD, SE, or 95% CI); and WTP threshold, K, were extracted. The cost-effective (CE) plane plotting ΔC and ΔE was also retrieved. Our intervention of interest was any GLP1, and the comparator was any other second-line antidiabetic agent. Two reviewers (BSB and YKG) independently extracted the data; any disagreement was resolved by consensus with the senior author (AT).

Risk of bias was assessed using the modified Economic Evaluations Bias checklist,^13^ considering overall biases (11 items) and biases from model-specific aspects, that is, structure (four items), data (six items), and internal consistency (one item). Each item was graded as yes, partly, unclear, no, or not applicable.

### Outcome of interest

The primary outcome of interest was INB,^14 16^ which was calculated as detailed in online supplementary appendix II. Since all monetary units were reported in different currencies (ie, US$, €, £, and ¥) and at different time points (years), they were converted to purchasing power parity (PPP), adjusted to US$ for the year 2017 before calculating INB (see example in online supplementary appendix II).

10.1136/bmjdrc-2019-001020.supp2 Supplementary data

### Data preparation

To calculate the INB and its variance, means along with dispersions (SD, SE, and 95% CI) of ΔC and ΔE were required. However, economic studies reported different parameters; therefore, five scenarios were designed to deal with data as follows.^9 10^

#### Scenario 1

Studies reported means, along with measures of dispersion for costs, outcomes, ΔC, ΔE, and ICER. The INB was estimated accordingly to equations (1), (2) and (3) as detailed in online supplementary appendix II.

#### Scenario 2

Studies reported ICER, along with its 95% CIs. The variance of ICER was calculated as $UL_{ICER}=\mu_{ICER}+1.96SE_{ICER}$. For model-based analyses, costs and QALYs were derived from simulated patients with infinite sample size; thus, the square of SD and SE were taken as the variance without considering the sample size. The mean and variance of INB were calculated using equations (2) and (3) in online supplementary appendix II.

#### Scenario 3

Studies reported means, along with any of 95% CI, SD or SE of costs, outcomes, or ΔC/ΔE, but did not provide the ICER and its variance. Data for ΔC and ΔE were then simulated using Monte Carlo with 1000 simulations. A gamma distribution was used for ΔC, and normal distribution was used for ΔE. The mean and variance of INB were then calculated using equations (1) and (4) in online supplementary appendix II.

#### Scenario 4

Studies did not report any measure of dispersion but provided the CE plane graph (ie, a scatter plot of ΔC and ΔE on Y and X axis) as part of a probabilistic sensitivity analysis (PSA) analysis. Data for ΔC and ΔE were extracted from the CE plane using WebPlotDigitizer software V.4.1.^15^ The mean of the ΔC and ΔE, along with their variances and covariances were estimated, leading to an estimated INB and its variance using equations (1) and (4) in online supplementary appendix II.

#### Scenario 5

Studies reported means of costs, outcomes, ΔC, ΔE, or ICER, but reported neither the dispersion nor the CE plane graph. The dispersions were taken from other studies that had reported/simulated data with the following criteria:

- Their ICERs were similar, for example, ±50% to 75%.
- They were similar in intervention, comparator, time period, and country region.
- They were in the same level of country income, with similar model inputs (ie, discounting and time horizon).
- If there was more than one study that met the criteria, the average of the variances of those studies was used.

### Statistical analysis

Each INB and its variance were calculated as per the approaches described previously. INBs were then pooled across studies stratified by low-income countries (LICs), lower-income to middle-income countries (LMICs), upper-income to middle-income countries (UMICs) and high-income countries (HICs) as per the World Bank classification.^8^ Meta-analysis was applied to pool the INBs^10 16^ using a random-effects model if heterogeneity was present (ie, I^2^≥25% or p value of Cochrane-Q<0.1); otherwise, a fixed-effects model was used (see more details in online supplementary appendix II). Sources of heterogeneity were explored using metaregression by considering covariables (ie, time horizon, discount rate, thresholds, and source of effectiveness measure) in the model one-by-one. If the I^2^ was decreased by 50% or more, such covariables were considered a source of heterogeneity. Sensitivity or subgroup analysis was performed where appropriate. Publication bias was assessed using funnel plots and Egger's test; any sources of asymmetry were explored using contour-enhanced funnel plots. All data were prepared using Microsoft Excel V.2016 and were analyzed by STATA software V.14.^17^ Two-sided p<0.05 was considered statistically significant except for the heterogeneity test, in which p<0.10 was used.

## Results

**Figure 1:**
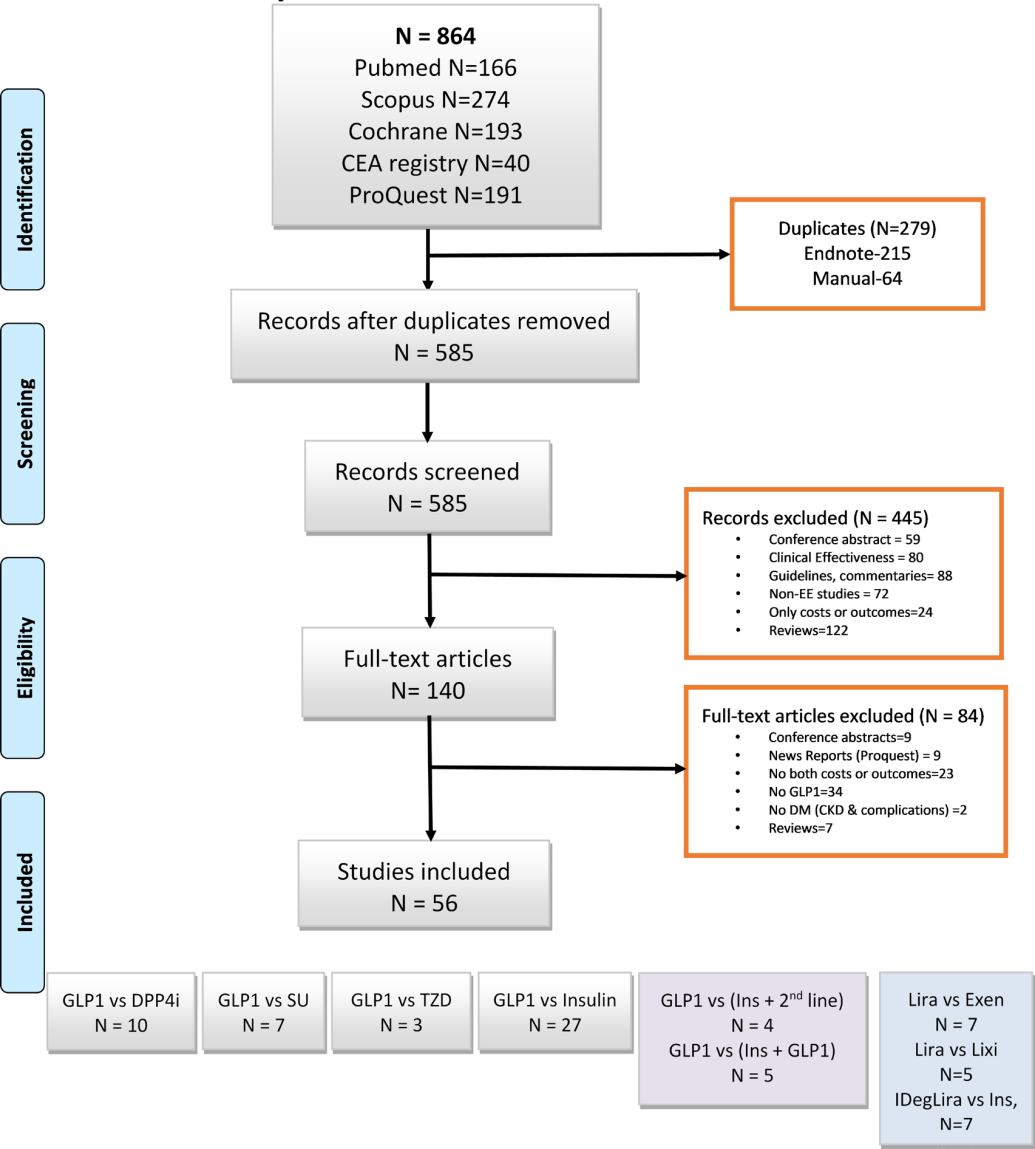
Study selection flow of GLP1 pooling. CEA, Cost-Effectiveness Analysis; CKD, chronic kidney disease; DM, diabetes mellitus; DPP4i, dipeptidyl peptidase-4 inhibitor; Exen, exenatide; EE, economic evaluation; GLP1, glucagon-like peptide 1; IDegLira, insulin degludec/liraglutide; Lira, liraglutide; Lixi, lixisenatide; SU, Sulfonylurea; TZD, thiazolidinedione.

Of the 864 identified studies, 56 studies were eligible for the meta-analysis (see figure 1). From the 56 studies, 82 comparisons were assessed, including GLP1 versus DPP4i (n=10)^18–27^; GLP1 versus sulfonylureas (n=7)^20 25–30^; GLP1 versus thiazolidines (n=3)^21 30 31^; GLP1 versus insulins (n=27, 23 HICs^19 30–52^ and 3 UMICs^53–55^); GLP1 versus insulin plus DPP4i (n=2),^45 56^ or insulin plus sulfonylureas (n=2),^52 57^ GLP1 versus insulin plus GLP1 (n=5)^36 42 46 55 58^ and insulin degludec/liraglutide (IDegLira) versus insulin (n=7).^34–36 42 46 48 59^ Among GLP1s, treatment comparisons included liraglutide versus exenatide (n=7)^43 56 60–64^ and liraglutide versus lixisenatide (n=5).^23 43 65–67^

Economic studies were mainly performed using a payer’s perspective, except for 10 comparisons (n=7^27 36 41 45 53 58 64^) that used a societal perspective (online supplementary table 1). The mean age of patients ranged from 50.9 to 64.7 years. Comparisons used models based on lifetime (n=27) or non-lifetime horizons (n=53); two had no time horizon information (n=2). Comparisons were mainly from HIC; only eight comparisons (n=7^28 53–55 57 62 64^) were from UMIC and none from LMIC/LIC. For the sources of model input parameters, the majority of comparisons (n=41) used single study-based estimates, followed by multiple study-based (n=28), synthesis-based (n=12) and hospital-based.^64^ The gross domestic product (GDP)-based thresholds were used for WTP in 12 comparisons (n=8^28 36 46 48 53–55 68^), and nine comparisons (n=5^21 26 31 52 64^) did not mention/unclear, and the rest used country-specific thresholds (online supplementary table 2). Sensitivity analysis was performed using PSA in all except 13 studies.^20 21 24 26 28 30 31 35 43 49 51 52 56^

10.1136/bmjdrc-2019-001020.supp3 Supplementary data

Four comparisons (n=3) reported that GLP1s were not cost-effective compared with insulin,^51^ sulfonylureas,^26^ DPP4i,^26^ and SGLT2 plus sulfonylureas^69^; four comparisons from a single study^30^ did not compare with any threshold; and the rest of GLP1 comparisons were cost-effective or superior relative to comparators (see online supplementary table 1).

Risk of bias assessment was performed (online supplementary figure 1). Most studies had a similar bias profile, except double counting and reporting/dissemination had unclear information. Four^35 51 52 57^ and 14 studies^21 24 28 30 35 36 49 51 52 54 57 61 66 69^ had bias related to model structures and data, respectively. All studies were unclear for internal consistency bias.

10.1136/bmjdrc-2019-001020.supp4 Supplementary data

### GLP1 versus DPP4i

**Figure 2:**
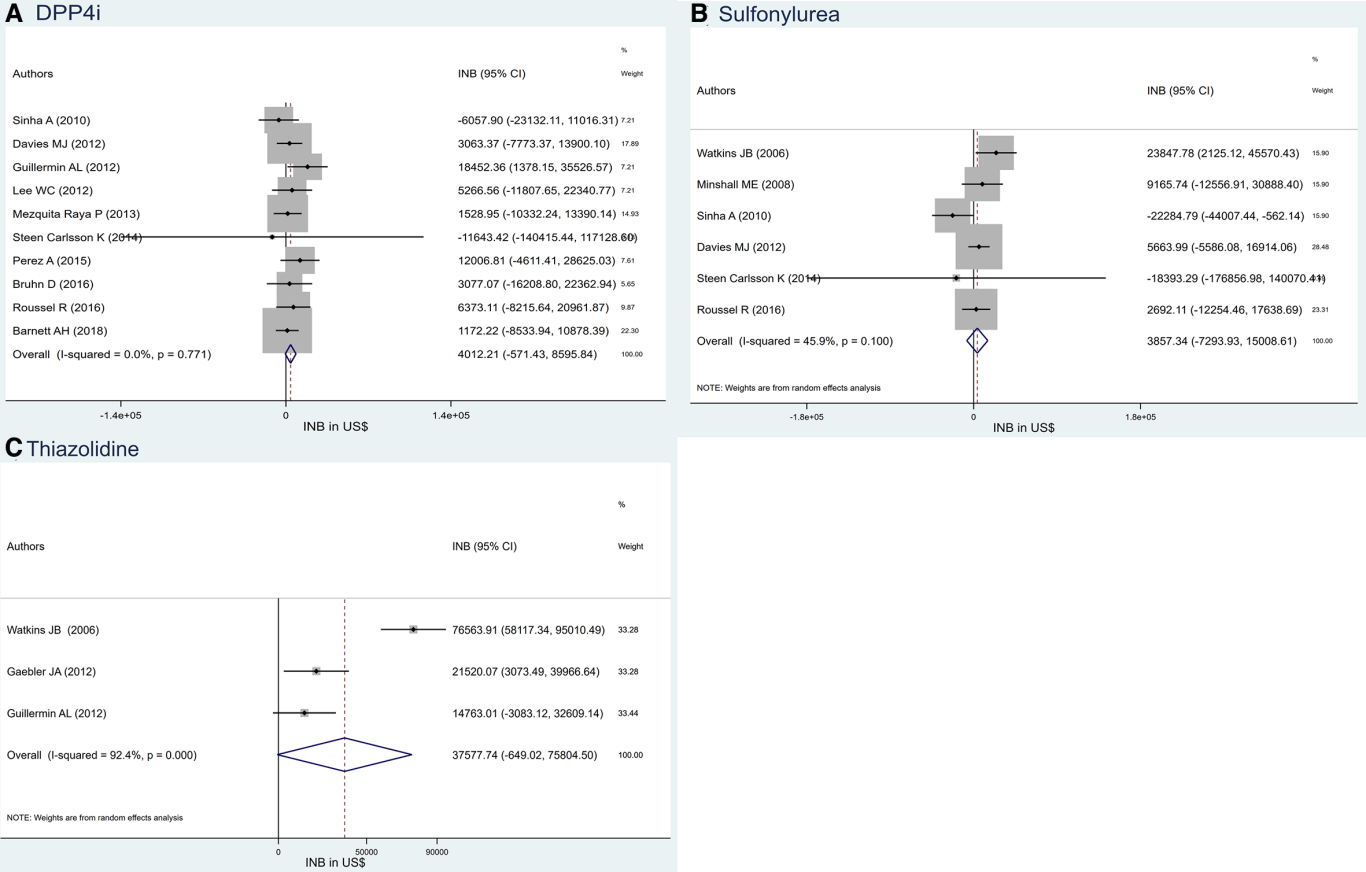
Pooling INB of glucagon-like peptide 1 versus (A) DPP4i, (B) sulfonylurea and (C) thiazolidines and its funnel plot. DPP4i, dipeptidyl peptidase-4 inhibitor; INB, incremental net benefit.

INBs of GLP1 versus DPP4i were estimated (n=10^18–27^), and all were from HICs with no heterogeneity (I^2^=0, figure 2A). The INB_p_ was US$4012.21 (95% CI US$−571.43 to US$8595.84), which favours GLP1 compared with DPP4i, but does not reach statistical significance.

The threshold used for these comparisons ranged from US$29 382 to US$58 024. A sensitivity analysis omitting the study that used a societal perspective^27^ and the study that did not use discounting^19^ yielded INB_p_s of US$4032.07 (95% CI US$−554.48 to US$8618.61) and US$4068.19 (95% CI US$−650.66 to US$8787.04), respectively (online supplementary figure 2A, B). These estimates are very similar to the main result and are robust.

In addition, subgroup analyses by WTP threshold (< vs ≥median of US $49 325), time horizon, and source of effectiveness measure were performed, indicating GLP1s were not significantly cost-effective compared with DPP4i in any subgroup (online supplementary figure 3A-C). There was no evidence of publication bias in either a funnel plot (online supplementary figure 4) or Egger’s test (coefficient=0.32, SE=0.73, p=0.672).

### GLP1 versus sulfonylureas

Seven studies^25–30 34^ compared GLP1 versus sulfonylureas, and all were conducted in HICs except one study.^28^ INBs of HIC were moderately heterogeneous (I^2^=45.9; see figure 2B) with an INB_p_ of US$3857.34 (95% CO US$−7293.93 to US$15 008.61), that is, again favouring GLP1 compared with sulfonylureas but not reaching statistical significance.

The threshold used varied from US$34 905 to US$62 757. Omitting two studies with the highest WTP threshold,^29 30^ societal perspective,^27^ or no discounting^30^ from overall pooling yielded INB_p_s of US$−1775.65 (95% CI US$−14 537.27 to 10 985.97), US$3947.85 (95% CI US$−7865.66 to US$15 761.36) and US$606.93 (95% CI −9647.87 to US$10 861.72), respectively (see online supplementary figure 5A-C). These INB_p_s were not significant; that is, GLP1 was not cost-effective compared with sulfonylureas. Subgroup analyses showed that GLP1s were cost-effective compared with sulfonylureas at thresholds ≥US$57 474 and with a non-life-time horizon (see online supplementary figure 6A−C). No evidence of publication bias was suggested by either a funnel plot (see online supplementary figure 7) or Egger’s test (coefficient=−0.32, SE=1.38, p=0.825).

### GLP1 versus thiazolidines

INBs of GLP1s versus thiazolidines from HICs^21 30 31^ showed high heterogeneity (I^2^=92.4; see figure 2C). The INB_p_ was US$37 577.74 (95% CI US$−649.02 to US$75 804.50), that is, again favouring GLP1 compared with thiazolidines but not reaching significance. There was some asymmetry in the funnel plot (see online supplementary figure 8), but with the small number of included studies, this was not reliable.

### GLP1 versus insulins

Among the studies looking at GLP1s versus insulins, 24 and 3^53–55^ were from HICs and UMICs. One study^51^ had an outlier INB (based on scenario 5) and was excluded. The INBs were highly heterogeneous (I^2^=86.4) with a pooled INB_p_ of US$14 062.42 (95% CI US$8168.69 to US$19 956.15) (see online supplementary figure 9A); that is, GLP1s were cost-effective compared with insulins in HICs.

The thresholds ranged from US$6411 to 103 418. Omitting studies with the highest^70^ and lowest WTP threshold,^33^ those without discounting,^30 31 35^ and those using a societal perspective^36 41 45^ from overall pooling resulted in INB_p_s of US$14 136.28 (95% CI US$8163.24 to US$20 109.32), US$14 954.40 (95% CI US$8434.54 to US$21 474.26), US$13 214.95 (US$6905.07 to US$19 524.82), and US$12 889.17 (95% CI US$7073.30 to US$18 705.05), respectively (see online supplementary figure 10A−D). The INB_p_s were robust for all conditions; that is, GLP1s were cost-effective compared with insulins in all sensitivity analyses. Subgroup analyses by median WTP threshold (US$ 52 359), time horizon, and source of effectiveness measure indicated GLP1s were significantly cost-effective in all subgroups (see online supplementary figure 11A−C). Funnel plots (online supplementary figure 12A) and Egger’s test (coefficient=1.76, SE=0.73, p=0.025) showed asymmetry. A contour-enhanced funnel plot was constructed (online supplementary figure 12B), suggesting that asymmetry may be due to both heterogeneity and missing studies in significant areas.

GLP1s were not cost-effective compared with insulins in UMICs (n=3^53–55^) with INB_p_ of US$35 372.19 (95% CI US$−9955.53 to US$80 899.91, I^2^=91.3%), see online supplementary figure 9B. A funnel plot and Egger’s test (coefficient=3.40, SE=0.07, p=0.013) showed asymmetry; a contour-enhanced funnel plot suggested that this may be due to both heterogeneity and missing studies (see online supplementary figure 13A, B).

### Liraglutide versus exenatide

**Figure 3:**
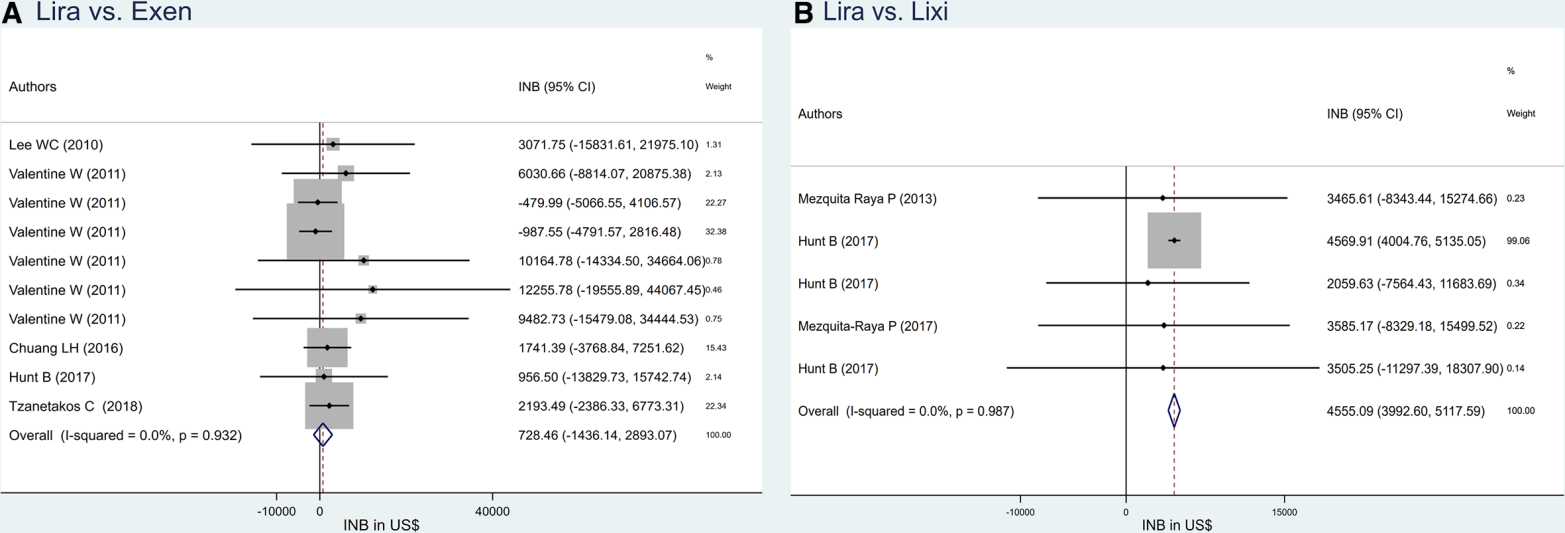
Pooling INB of Lira versus (A) Exen and (B) Lixi. Exen, exenatide; INB, incremental net benefit; Lira, liraglutide; Lixi, lixisenatide.

Among 12 comparisons (n=7^43 56 60–64^) of liraglutide versus exenatide, 10 comparisons (n=5) from HICs were pooled with no heterogeneity (I^2^=0). The INB_p_ was US $728.46 (95% CI US$−1436.14 to US$2893.07) (see figure 3A); that is, liraglutide was not more cost-effective compared with exenatide. Omitting studies with exenatide plus sulfonylureas,^61 62^ or highest and lowest WTP threshold^63^ from overall pooling, yielded INB_p_s of US$697.33 (95% CI US$−1481.61 to US$2876.27), US$674.84 (95% CI US$−1494.79 to US$2844.48) and US$1550.17 (95% CI US$−1082.16 to US$4182.50), respectively (see online supplementary figure 14A−C), indicating that liraglutide was not more cost-effective compared with exenatide.

Liraglutide was not more cost-effective in subgroups of WTP threshold (median US$50 967) or source of effectiveness (see online supplementary figure 15A, B). A funnel plot (see online supplementary figure 16A) and Egger’s test (coefficient=0.78, SE=0.24, p=0.01) suggested asymmetry; a contour-enhanced funnel plot further suggested that this may be due to missing studies in significant areas (see online supplementary figure 16B).

### Liraglutide versus lixisenatide

INBs of liraglutide versus lixisenatide were estimated in HICs^23 43 65–67^ with no heterogeneity (I^2^=0). The INB_p_ was US$4555.09 (95% CI US$3992.60 to US$5117.59); that is, liraglutide was significantly more cost-effective compared with lixisenatide (see figure 3B).

Omitting highest and lowest WTP thresholds^43^ yielded INB_p_s of US$4556.61 (95% CI US$3993.71 to US$5119.51) and US$4563.65 (95% CI US$4000.19 to US$5127.11), respectively (see online supplementary figure 17A, B); that is, the INB_p_s were robust. There was no evidence of publication bias by either funnel plot (online supplementary figure 18) or Egger’s test (coefficient=−0.26, SE=0.09, p=0.069).

### GLP1s versus insulin plus second-line drugs

INBs of GLP1s versus insulin plus other second-line agents (ie, insulin plus DPP4i^45 56^ and insulin plus sulfonylureas^52 57^) from HICs were pooled (I^2^=0) with INB_p_ of US$2071.10 (95% CI US$−10 355.78 to US$14 497.99, online supplementary figure 19A), indicating that GLP1 was not cost-effective compared with insulin plus other second-line agents. There was no evidence of publication bias on Egger’s test (coefficient=−1.75, SE=0.53, p=0.189).

The INB_p_ of GLP1s versus insulin plus GLPs from four HICs^36 42 46 55 58^ was US$20 509.08 (95% CI US$5435.21 to US$35 582.94, online supplementary figure 19B), indicating GLP1s were significantly more cost-effective compared with insulin plus GLP1s.

Omitting the studies with the highest WTP threshold and with a lifetime horizon^46^ resulted in INB_p_ of US$19 913.92 (95% CI US$−2496.45 to US$42 324.29) and US$26 396.17 (95% CI US$9067.27 to US$43 725.07), respectively (see online supplementary figure 20A, B). GLP1s were no longer cost-effective compared with insulin plus GLP1s after omitting the highest threshold study. There was no evidence of publication bias on funnel plot (online supplementary figure 21) or Egger’s test (coefficient=-1.49, SE=4.57, p=0.775).

### IDegLira versus insulin

INBs of IDegLira versus insulin from HICs^34–36 42 46 48 59^ showed high heterogeneity (I^2^=87.6). The INB_p_ was US $15 649.28 (95% CI US$3748.17 to US$27 550.39); that is, IDegLira was more cost-effective compared with insulin (see online supplementary figure 19C).

Omitting the highest^70^ and lowest WTP thresholds^59^ studies and the one using a societal perspective^36^ from overall pooling resulted in INB_p_s of US$16 078.96 (95% CI US$3537.45 to US$28 620.46), $15 440.64 (95% CI US$2091.91 to US$28 789.38), and US$5164.81 (95% CI US$1129.32 to US$9200.30), respectively (see online supplementary figure 22A−C). The INB_p_s were robust; that is, IDegLira was still cost-effective. In addition, IDegLira was still cost-effective in subgroups based on time horizon and source of effectiveness, but not in WTP<US$42 049 (see online supplementary figure 23A−C). There was no evidence of publication bias in the funnel plot (online supplementary figure 24) or Egger’s test (coefficient=2.79, SE=1.86, p=0.194).

## Discussion

**Figure 4:**
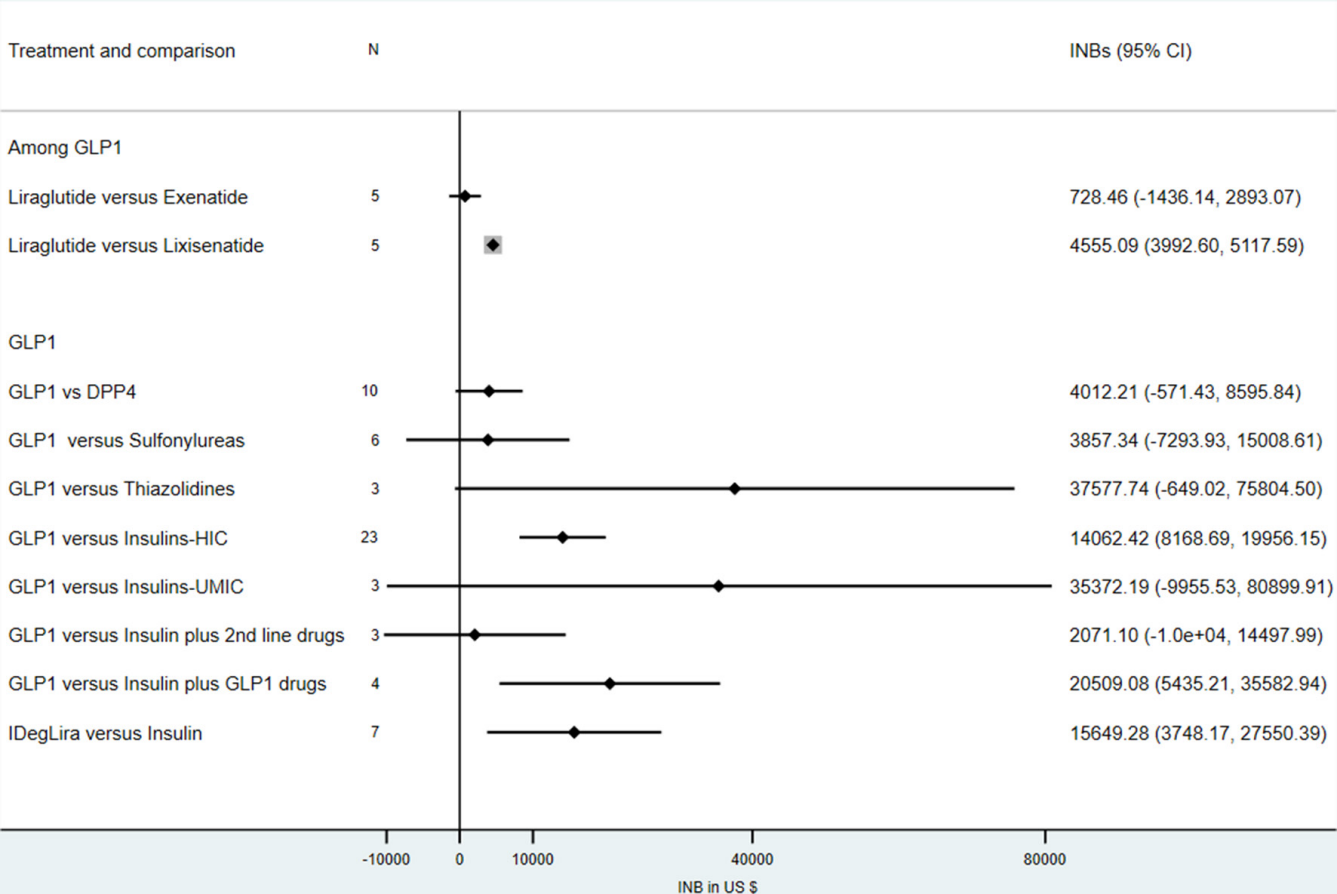
INB summary of GLP1 comparisons. GLP1, glucagon-like peptide 1; HIC, high-income countries; IDegLira, insulin degludec/liraglutide; INB, incremental net benefit; UMIC, upper-income to middle-income countries.

Meta-analysis of economic studies suggests that GLP1s are significantly more cost-effective compared with insulin in HICs but not in UMICs (figure 4); point estimates versus other antidiabetic agents, including DPP4i, sulfonylureas, and thiazolidines, also favour GLP-1, but these did not reach statistical significance. In addition, GLP1s are significantly cost-effective compared with insulin plus GLP1s, but not for insulin plus other second-line drugs. Furthermore, among GLP1 agonists, liraglutide was significantly more cost-effective compared with lixisenatide but not exenatide. Lastly, IDegLira was also significantly more cost-effective compared with insulin.

The GLP1s were cost-effective compared with insulins, mainly in HICs but not in UMICs. Although results had highly heterogeneity, they were very robust across multiple sensitivity and subgroup analyses, although there was some indication of publication bias. GLP1s were not cost-effective compared with insulin in UMICs, but this was based on only three studies.

The GLP1s were not cost-effective compared with DPP4i, sulfonylureas, and thiazolidines with varying degrees of heterogeneity (0%–92.7%). Interestingly, GLP1s were cost-effective in a subgroup analysis of sulfonylureas with respect to time horizon (lifetime vs non-lifetime), indicating that GLP1s would be cost-effective in the short term rather than the long term. In other words, DPP4i, sulfonylureas, and thiazolidines could be the drug of choice in the long term, but GLP1s might be a better choice in the short-term.

Results also suggested that treatment with GLP1s alone was significantly more cost-effective than insulin plus GLP1s, but not when compared with insulin plus second-line agents. This suggests that the combination of GLP1 with insulin might not be a good choice, but combining insulin with another second-line drug (eg, DPP4i and sulfonylureas) might be beneficial. This is consistent with our finding that IDegLira, a fixed-dose combination of insulin and degludec/liraglutide, was cost-effective compared with only insulin therapy.

Most findings of these economic studies relied on the point estimate ICER (ie, deterministic ICER) for decision making while ignoring the measures of dispersion. This meta-analysis considered ranges of ICERs rather than the point estimates. This study standardized monetary units by converting them to 2017 $US using the Consumer Price Index (CPI) and PPP conversions, which provided reliability of cost-effectiveness for the most recent time point considering a country’s economic changes across time. In addition, PPP adjustment provides values for comparison across the globe, even when considering different worldwide economic conditions and time-lag adjustments.

Although many previous reviews of economic evaluations of T2DM treatments have been performed,^71–77^ these were narrative reviews,^72 73 77^ or systematic reviews^71 72 74 78 79^ without synthesis of economic outcomes, or focused on cost/burden of illness.^73–76^ Our study reported pooled economic results that were adjusted with PPP and time-lag and standardized across countries.

### Strengths and limitations

Our study had significant strengths. Given that economic evaluation studies varied in reporting results, we calculated and pooled a common economic parameter using meta-analysis stratified by country income, which has rarely been performed before. Five scenarios were constructed to calculate this common parameter and standardize it before pooling. The different monetary units were all converted to a common standard currency. We used INB instead of ICER as the economic effect measure because of limitations of the ICER.^80^ For instance, a negative ICER may indicate a lower cost compared with higher effectiveness of intervention or higher cost, along with lower effectiveness of intervention, thus introducing ambiguity in interpretation^16 81^; in contrast, positive and negative INBs directly indicate cost-effectiveness and non-cost-effectiveness, which is the information required by policy makers.^82 83^ Such information helps provide evidence informed policy for decision makers from both resource-rich and resource-poor countries.

Another challenge for pooling economic studies was heterogeneity, caused by study design (model or alongside clinical trials), population, country, GDP, or economic perspective taken. The CPI and PPP were applied to standardize different economic backgrounds, as well as the time lag across the studies.^84 85^ However, it should be noted that using PPP may have some limitation related to the method of parity estimation as price indices are calculated from individual prices of only selected commodities rather than all commodities in each country.^86^ In addition, results from economic studies depend greatly on important factors such as WTP thresholds, analytical time horizons, country income or GDP, effectiveness measurement used, discount rates, and perspectives. These factors were taken into account by performing stratified analyses by level of country income, subgroup analyses, and sensitivity analyses where appropriate.

Our study also has some limitations. These findings apply largely to HICs. Although we used five scenarios to estimate variances of the outcome measure (ie, ICER), we could not assess validity of data, particularly for scenarios 3–4 from Monte Carlo simulation or extraction of data from the CE plane by WebPlotDigitizer due to lack of actual or raw data obtained from these studies. In addition, it should be noted that individual countries differ in T2DM prevalence, patient behaviors, treatment regimens and accessibility, and healthcare systems. Therefore, all these factors should be considered when applying our findings.

Future primary studies may be directed towards bridging the current knowledge gap in terms of studies from LIC and LMIC economies, inclusion of other antidiabetic drug groups for comparison, as well as by following a standard reporting format for economic studies, that is, reporting the measures of dispersion of the study point estimates and reporting the INB rather than just ICER. In terms of meta-analysis, further fine-tuning and standardization of meta-analysis of economic evaluation study methods is essential mainly in terms of standardization of data-extraction methods/guidelines in economic studies, and assessment of heterogeneity and publication. In addition, future studies may explore the feasibility of conducting a network meta-analysis of economic studies.

## Conclusion

In HICs, GLP1s and IDegLira appear to be more cost-effective than insulins, but not DPP4i, sulfonylureas, and thiazolidines. Liraglutide appears to be more cost-effective compared with lixisenatide but not exenatide. Further primary economic evaluation studies in LICs and UMICs are required to address gaps in the literature.
