# Supplementary material for: Glucagon-like peptide 1 agonists for treatment of patients with type 2 diabetes who fail metformin monotherapy: systematic review and meta-analysis of economic evaluation studies
Source: BMJ Open Diabetes Res Care. 2020 Jul 19;8(1):e001020. doi: 10.1136/bmjdrc-2019-001020 (PMC7371226; doi:10.1136/bmjdrc-2019-001020)
Supplement: Supplementary data [file bmjdrc-2019-001020supp001.pdf]

APPENDIX I
SEARCH STRATEGIES

The search strategy:

The search terms were constructed based on domains of population, intervention, comparator, and outcome (PICO) as below. Then these search terms were combined using Boolean operator OR within the same domains, and "AND" Boolean operator between domains of PICO as described

- **Problem/Patient/Population**: Adult Type 2 diabetes mellitus patient.
  - (New classification: Severe Insulin-resistant diabetes (SIRD), Mild obesity-related diabetes (MOD), Mild age-related diabetes (MARD))
- **Intervention:** use of
  - GLP1 receptor agonists (Exenatide, Liraglutide, Albiglutide, Lixisenatide, Dulaglutide)
- **Comparator**: placebo or other anti-DM Drugs (Sulfonylureas, Meglitinides, Thiazolidinediones, α-Glucosidase inhibitors, DPP-4 inhibitors, Insulin)
- **Outcome**: Costs, Life Years, Quality Adjusted Life Years, Incremental Cost-Effective Ratios, Incremental Net Benefit.

1. Medline-PUBMED search

**Search**	**Builder**	**Query: Diabetes**
1	#1	Search "Diabetes Mellitus, Type 2"[Mesh]
2	#2	Search "Severe Insulin-resistant diabetes"
3	#3	Search SIRD
4	#4	Search "Mild obesity-related diabetes"
5	#5	Search "Mild age-related diabetes"
6	#1 OR #2 OR #3 OR #4 OR #5	Search (((("Diabetes Mellitus, Type 2"[Mesh]) OR "Severe Insulin-resistant diabetes") OR SIRD) OR "Mild obesity-related diabetes") OR "Mild age-related diabetes"
**Search**	**Builder**	**Query GLP**
1.		Search "glucagon-like peptide-1(28-36) amide" [Supplementary Concept]
2.		Search "glp1 agonists"
3.		Search "glucagon Like peptide 1 agonist$"
4.		Search exenatide
5.		Search bydureon
6.		Search byetta
7.	4 OR 5 OR 6	Search ((exenatide) OR bydureon) OR byetta
8.		Search liraglutide

9.		Search Victoza
10.		Search saxenda
11.	8 OR 9 OR 10	Search ((liraglutide) OR Victoza) OR saxenda
12.		Search albiglutide
13.		Search eperzan
14.		Search tanzeum
15.	12 OR 13 OR 14	Search ((albiglutide) OR eperzan) OR tanzeum
16.		Search lixisenatide
17.		Search lyxumia
18.		Search adlyxin
19.	16 OR 17 OR 18	Search ((lixisenatide) OR lyxumia) OR adlyxin
20.		Search dulaglutide
21.		Search trulicity
22.	20 OR 21	Search (dulaglutide) OR trulicity
23.	1 OR 2 OR 3 OR 7 OR 11 OR 15 OR 19 OR 22	Search (((((((("glucagon-like peptide-1(28-36) amide" [Supplementary Concept]) OR "glp1 agonists") OR "glucagon Like peptide 1 agonist$") OR (((exenatide) OR bydureon) OR byetta)) OR (((liraglutide) OR Victoza) OR saxenda)) OR (((albiglutide) OR eperzan) OR tanzeum)) OR (((lixisenatide) OR lyxumia) OR adlyxin)) OR ((dulaglutide) OR trulicity)
**Search**	**Builder**	**Query Economic Outcome**
1.		Search costs
2.		Search effectiveness
3.		Search ("Costs and Cost Analysis"[Mesh])
4.		Search "Cost-Benefit Analysis"[Mesh]
5.		Search "Economics"[Mesh]
6.		Search "Quality-Adjusted Life Years"[Mesh]
7.		Search "Quality-Adjusted Life Years"
8.		Search qaly
9.	7 OR 8	Search ("Quality-Adjusted Life Years") OR qaly
10.	6 OR 9	Search ("Quality-Adjusted Life Years"[Mesh]) OR (("Quality-Adjusted Life Years") OR qaly)
11.		Search ly
12.		Search "life years"
13.	11 OR 13	Search (ly) OR "life years"
14.		Search “incremental cost effectiveness ratio”
15.		Search ICER
16.	14 OR 15	Search (“incremental cost effectiveness ratio”) OR ICER
17.		Search "incremental net benefit”
18.		Search INB
19.	17 OR 18	Search ("incremental net benefit”) OR INB
20.	1 OR 2 OR 3 OR 4 OR 5 OR 10 OR 13 OR 16 OR 19	Search ((((((((costs) OR effectiveness) OR (("Costs and Cost Analysis"[Mesh]))) OR "Cost-Benefit Analysis"[Mesh]) OR "Economics"[Mesh]) OR (("Quality-Adjusted Life Years"[Mesh]) OR (("Quality-Adjusted Life Years") OR qaly))) OR ((ly) OR "life years")) OR ((“incremental cost effectiveness ratio”) OR ICER)) OR (("incremental net

		benefit") OR INB)

All Combined

Search	Query
1.	Query Diabetes #6
2.	Query GLP1 #23
3.	Query Economic Outcome #20
4.	#1 & #2 & #3

2. Search Strategy in Scopus:

1	ALL (diabetes AND mellitus)
2	ALL ("glp1 agonists" OR "glucagon Like peptide 1 agonist$" OR exenatide OR bydureon OR byetta OR liraglutide OR victoza OR saxenda OR albiglutide OR eperzan OR tanzeum OR lixisenatide OR lyxumia OR adlyxin OR dulaglutide OR trulicity)
3	ALL ("cost effectiv*" OR "Cost utility" OR "Cost benefit" OR "Cost-Benefit" OR "Quality Adjusted Life Years" OR qaly OR ly OR "life year$" OR "incremental cost effective ratio" OR "ICER" OR "incremental net benefit" OR inb)
4	#1 & #2 & #3

3. Search strategy in Cochrane:

Sl no	Search	Search terms
1		type 2 diabetes
2		(exenatide OR liraglutide OR albiglutide OR lixisenatide OR dulaglutide)
3		(costs OR effectiveness OR utility OR QALY OR Life Years OR incremental cost effectiveness ratio OR ICER)
8	**1 & 2 & 3**	
