# Supplementary material for: Glucagon-like peptide 1 agonists for treatment of patients with type 2 diabetes who fail metformin monotherapy: systematic review and meta-analysis of economic evaluation studies
Source: BMJ Open Diabetes Res Care. 2020 Jul 19;8(1):e001020. doi: 10.1136/bmjdrc-2019-001020 (PMC7371226; doi:10.1136/bmjdrc-2019-001020)
Supplement: Supplementary data [file bmjdrc-2019-001020supp002.pdf]

Appendix II

A) Estimation of incremental net benefit

Incremental net benefit (INB)[1,2] can be estimated as follows:

$$\mathrm{INB} = \mathrm{K} \times \Delta\mathrm{E} - \Delta\mathrm{C} \text{ ------------------------(1)}$$

or

$$\mathrm{INB} = \Delta\mathrm{E}\,(\mathrm{K} - \mathrm{ICER}) \text{ ------------------------(2)}$$

$$\mathrm{var(INB)} = \mathrm{K}^2\sigma^2_{\Delta\mathrm{E}} + \sigma^2_{\mathrm{ICER}} \text{---------------------(3)}$$

or

$$\mathrm{var(INB)} = \mathrm{K}^2\sigma^2_{\Delta\mathrm{E}} + \sigma^2_{\Delta\mathrm{C}} - 2\mathrm{K}\rho_{\Delta\mathrm{C}\Delta\mathrm{E}} \text{ --------(4)}$$

where K was the WTP, ΔC and ΔE were incremental cost and incremental effective, $\sigma^2_{\Delta C}, \sigma^2_{\Delta E}, \rho_{\Delta C \Delta E}$ were variances of ΔC and ΔE and covariance theiroff, and σ^2_{ICER} was variance of ICER . The WTP was used as reported in the original included studies, i.e., a standard/country specific or GDP based WTP threshold. A positive INB favours treatment, i.e., intervention is cost-effective, whereas a negative INB favours the comparator, i.e., intervention is not cost-effective[1,3,4].

Currency conversions and standardization

The monetary units were converted to purchasing power parity (PPP), adjusted to US$ for the year 2017 before INB calculation. For instance, if a study reported cost, ICER, and thresholds in Euros for 2012, this currency was first converted to 2017 Euros using the historical consumer price index (CPI) of that country. The Euro 2017 value was next converted to PPP adjusted US$ rate using conversion rates from the International Monetary Fund[5]. In addition, the K value from GDP-based threshold was corrected for both latest CPI (2017) and PPP, while for standard/country specific or fixed K, only PPP was corrected. For the variance monetary value conversion, the specific study variance was multiplied by the square of total factors (i.e., CPI and PPP) for the year 2017. For example, if Y is variance of ICER in Euros 2012, this was converted into 2017 PPP adjusted US$ as

$$\mathrm{Y_{ppp2017}} = \mathrm{Y_{€2012}} \times \left(\frac{\mathrm{cpi_{€2017}}}{\mathrm{cpi_{€2012}}} \times \frac{1}{\mathrm{ppp_{2017}}}\right)^2 \text{ ---------------(5)}$$

B) Meta-analysis

i) A fixed-effects model

$$\mathrm{INB_p} = \frac{\sum_{\mathrm{i=1}}^{\mathrm{S}} \mathrm{w_i . INB_i}}{\sum_{\mathrm{i=1}}^{\mathrm{S}} \mathrm{w_i}} \text{----------------------(1)}$$

$$w_i = \frac{1}{Var(INB_i)} \text{----------------------------(2)}$$

ii) A random-effects model

$$\mathrm{INB_p} = \frac{\sum_{\mathrm{i=1}}^{\mathrm{S}} w_i^* \mathrm{.INB_i}}{\sum_{\mathrm{i=1}}^{\mathrm{S}} w_i^*} \text{----------------------(3)}$$

$$w_i^* = \frac{1}{Var(INB_i)+\tau^2} \text{-----------------------(4)}$$

$$\tau^2 = \frac{Q-(S-1)}{\sum w_i - \frac{\sum w_i^2}{\sum w_i}} \text{----------------------------(5)}$$

Q is the Cochrane Q-statistic, where Q = 0 if Q < S-1; and s is the number of included studies/comparisons.

The heterogeneity of INB was assessed using Cochrane Q-test and I^2 statistic calculated as equations below.

$$\mathrm{Q} = \sum_{\mathrm{i-1}}^{\mathrm{S}} \mathrm{w_i} \left(\mathrm{INB_i} - \mathrm{INB}_p\right)^2 \text{-------------(6)}$$

$$\mathrm{I}^2 = 100\%\mathrm{x}\ \frac{\mathrm{Q-(S-1)}}{\mathrm{Q}} \text{----------------------(7)}$$

1. Crespo C, Monleon A, Diaz W, Rios M. Comparative efficiency research (COMER): meta-analysis of cost-effectiveness studies. *BMC Med Res Methodol.* 2014;14:139.
2. Stinnett AA, Mullahy J. Net health benefits: a new framework for the analysis of uncertainty in cost-effectiveness analysis. *Med Decis Making.* 1998;18(2 Suppl):S68-80.
3. Willan AR, Lin DY. Incremental net benefit in randomized clinical trials. *Stat Med.* 2001;20(11):1563-1574.
4. Willan AR. Incremental net benefit in the analysis of economic data from clinical trials, with application to the CADET-Hp trial. *Eur J Gastroenterol Hepatol.* 2004;16(6):543-549.
5. IMF data. https://www.imf.org/external/pubs/ft/weo/2018/01/weodata/index.aspx. Accessed 2018-12-03, 2018.
