# Supplementary material for: Glucagon-like peptide 1 agonists for treatment of patients with type 2 diabetes who fail metformin monotherapy: systematic review and meta-analysis of economic evaluation studies
Source: BMJ Open Diabetes Res Care. 2020 Jul 19;8(1):e001020. doi: 10.1136/bmjdrc-2019-001020 (PMC7371226; doi:10.1136/bmjdrc-2019-001020)
Supplement: Supplementary data [file bmjdrc-2019-001020supp003.pdf]

Suppl. Table 1 Characteristics of selected studies for GLP1 pooling

Author	Country	(yr) Age	Men	(yr) TH	Model	DC	DE	Study type	Intervention	Comparison	GLP1 CE
Watkins JB 2006**[39]**	USA	53	50	30	CDM	NM	NM	Single study	Exen	SU	CE
Watkins JB 2006**[39]**	USA	53	50	30	CDM	NM	NM	Single study	Exen	TZD	CE
Watkins JB 2006**[39]**	USA	53	50	30	CDM	NM	NM	Single study	Exen	Ins	CE
Watkins JB 2006**[39]**	USA	53	50	30	CDM	NM	NM	Single study	Exen	Placebo	CE
Ray JA 2007**[58]**	UK	58.9	55.7	35	CDM	3.5	3.5	Single study	Exen	Ins	CE
Minshall ME 2008**[38]**	USA	56	63	30	CDM	3	3	Multi-study	Exen	SU	CE
Woehl A 2008**[60]**	UK	59	56	40	DES	3.5	3.5	Multi-study	Exen	Ins	not CE
Brandle M 2009**[42]**	Switzerland	58.9	55.7	35	Not clear	2.5	2.5	Single study	Exen	Ins	CE
Mittendorf T 2009**[56]**	Germany	58.9	55.7	10	IMS	5	5	Single study	Exen	Ins	CE
Lee WC 2010**[70]**	USA	56.7	51.9	35	IMS	3	3	Single study	Lira	Exen+SU	CE
Sinha A 2010**[35]**	USA	NM	NM	Life	CDC	3	3	Multi-study	Exen	SU	not CE
Sinha A 2010**[35]**	USA	NM	NM	Life	CDC	3	3	Multi-study	Exen	DPP4i	not CE
Beaudet A 2011**[41]**	UK	58	53.3	50	IMS	3.5	3.5	Single study	Exen	Ins	CE
Goodall G 2011**[47]**	Spain	57.37	54.1	35	IMS	3	3	Single study	Exen	Ins	CE
Ivanova A 2011**[66]**	Bulgaria	NM	NM	20	IMS	NM	NM	Single study	Lira	Ins+SU	CE
Lee W 2011**[85]**	USA	56.1	49.4	35	IMS	3	3	Single study	Lira	SU+TZD	CE
Petrova G 2011**[71]**	Bulgaria	NM	NM	20	IMS	5	3	Single study	Lira	Exen+SU	Lira CE
Valentine W 2011**[72]**	Switzerland	56.7	51.9	40	IMS	3	3	Single study	Lira	Exen	CE
Valentine W 2011**[72]**	Denmark	56.7	51.9	40	IMS	3	3	Single study	Lira	Exen	CE
Valentine W 2011**[72]**	Norway	56.7	51.9	40	IMS	4	4	Single study	Lira	Exen	CE

Suppl.Table 1 Characteristics of selected studies for GLP1 pooling (cont.)

Author	Country	(yr) Age	Men	(yr) TH	Model	DC	DE	Study type	Intervention	Comparison	GLP1 CE
Valentine W 2011**[72]**	Finland	56.7	51.9	40	IMS	3	3	Single study	Lira	Exen	CE
Valentine W 2011**[72]**	Netherland	56.7	51.9	40	IMS	4	1.5	Single study	Lira	Exen	CE
Valentine W 2011**[72]**	Austria	56.7	51.9	40	IMS	3.5	3.5	Single study	Lira	Exen	CE
Davies MJ 2012**[29]**	UK	55.8	54.2	Life	IMS	3.5	3.5	Single study	Lira	SU	CE
Davies MJ 2012**[29]**	UK	55.3	52.9	Life	IMS	3.5	3.5	Single study	Lira	DPP4i	CE
Gaebler JA 2012**[40]**	USA	57.3	51.3	20	TAM	none	none	Multi-study	Exen	TZD	dominant
Gaebler JA 2012**[40]**	USA	57.3	51.3	20	TAM	none	none	Multi-study	Exen	Ins	dominant
Gao L 2012**[37]**	China	NM	NM	30	UKPDS	3	3	Multi-study	Lira	SU	CE
Guillermin AL 2012**[30]**	USA	52.5	51.7	35	IMS	3	3	Single study	Exen	DPP4i	dominant
Guillermin AL 2012**[30]**	USA	52.5	51.7	35	IMS	3	3	Single study	Exen	TZD	dominant
Lee WC 2012**[31]**	USA	55.3	52.9	35	IMS	3	3	Single study	Lira	DPP4i	CE
Samyshkin Y 2012**[59]**	USA	57.9	53.3	35	IMS	3	3	Single study	Exen	Ins	CE
Fonseca T 2013**[46]**	Spain	54.75	57	35	IMS	3	3	Multi-study	Exen	Exen	EQW CE
Fonseca T 2013**[46]**	Spain	54.75	57	35	IMS	3	3	Multi-study	Exen	Ins	EQW CE
Mezquita Raya P 2013**[32]**	Spain	55.3	52.9	Life	IMS	3	3	Single study	Lira	DPP4i	CE
Mezquita Raya P 2013**[32]**[*]	Spain	NM	NM	Life	IMS	3	3	Single study	Lira	Lixi	CE
Kiadaliri AA 2014**[54]**[*]	Sweden	64.7	57.5	35	SIHECM	3	3	Single study	GLP1	Ins+DPP4i	CE
Kiadaliri AA 2014**[54]**[*]	Sweden	NM	NM	35	SIHECM	3	3	Single study	GLP1	Ins	CE
Steen Carlsson K 2014**[36]**[*]	Sweden	57	NM	Life	IHE	3	3	Multi-study	Lira	SU	CE
Steen Carlsson K 2014**[36]**[*]	Sweden	56	NM	Life	IHE	3	3	Multi-study	Lira	DPP4i	CE

Suppl.Table 1 Characteristics of selected studies for GLP1 pooling (cont.)

Author	Country	(yr) Age	Men	(yr) TH	Model	DC	DE	Study type	Intervention	Comparison	GLP1 CE
Tzanetakos C 2014**[65]**	Greece	55.3	52.9	Life	IMS	3.5	3.5	Single study	Lira	Ins+DPP4i	CE
Zhang Y 2014**[61]**	USA	54.4	NM	NM	Markov	NM	NM	Multi-study	GLP1	Ins+SU	dominant
Zhang Y 2014**[61]**	USA	54.4	NM	NM	Markov	NM	NM	Multi-study	GLP1	Ins	dominant
Deng J 2015**[62]**[*]	China	51.74	49	40	Cardiff	3	3	Multi-study	Exen	Ins	CE
Huetson P 2015**[50]**[*]	Norway	57.2	46.1	45	IMS	4	4	Single study	Lixi	Ins	CE
Perez A 2015**[33]**	Spain	NM	NM	Life	IMS	3	3	Single study	Lira	DPP4i	CE
Bruhn D 2016**[28]**	USA	55.6	47.3	50	IMS	NM	NM	Multi-study	Albi	Ins	CE
Bruhn D 2016**[28]**	USA	54.5	47.6	50	IMS	NM	NM	Multi-study	Albi	DPP4i	CE
Davies MJ 2016**[43]**	UK	56.8	56.3	40	IMS	3.5	3.5	Synthesis based	IDegLira	Ins	CE
Gordon J 2016**[48]**	Sweden	59.8	50	Life	Cardiff	3	3	Single study	Exen	Ins	CE
Roussel R 2016**[34]**	France	55.3	52.9	Life	IMS	3	3	Multi-study	Lira	DPP4i	CE
Roussel R 2016**[34]**	France	55.8	54.2	Life	IMS	3	3	Multi-study	Lira	SU	CE
Zhang X 2016**[73]**[*]	China	52.3	57.2	30	IMS	3	3	Hospital data base	Lira	Exen	Lira CE
Dilla T 2017**[86]**	Spain	55.79	46.4	Life	IMS	3	3	Synthesis based	Dula	Lira	Dula CE
Ericsson A 2017**[45]**[*]	Sweden	57.2	NM	40	SIHECM	3	3	Multi-study	IDegLira	Ins	CE
Ericsson A 2017**[45]**[*]	Sweden	57.2	NM	40	SIHECM	3	3	Multi-study	IDegLira	Ins+GLP	CE
Hunt B 2017**[68]**	Netherland	56.8	56.3	Life	IMS	4	1.5	Single study	IDegLira	Ins	CE
Hunt B 2017**[75]**	Italy	NM	NM	Life	IMS	3	3	Single study	Lira	Lixi	CE
Hunt B 2017**[51]**	USA	NM	NM	50	IMS	3	3	Synthesis based	IDegLira	Ins+GLP	IDegLira dominant
Hunt B 2017**[79]**	USA	58.8	50.3	50	IMS	3	3	Single study	IDegLira	Ins	CE

Suppl.Table 1 Characteristics of selected studies for GLP1 pooling (cont.)

Author	Country	(yr) Age	Men	(yr) TH	Model	DC	DE	Study type	Intervention	Comparison	GLP1 CE
Hunt B 2017**[74]**	UK	NM	NM	not clear	IMS	3.5	3.5	Single study	Lira	Lixi	Lira CE
Kvapil M 2017**[55]**	Czech Republic	56.8	56.3	Life	IMS	3	3	Multi-study	IDegLira	Ins	CE
Kvapil M 2017**[55]**	Czech Republic	56.8	56.3	Life	IMS	3	3	Multi-study	IDegLira	Ins+GLP	CE
Lasalvia P_2017**[64]**	Columbia	NM	NM	5	Markov	5	5	Multi-study	Dula	Ins	CE
Lasalvia P_2017**[64]**	Columbia	NM	NM	5	Markov	5	5	Multi-study	Dula	Ins+GLP	dominant
Mezquita-Raya P 2017**[76]**	Spain	NM	NM	Life	IMS	3	3	Single study	Lira	Lixi	Lira CE
Psota M 2017**[57]**	Slovakia	NM	NM	Life	IMS	5	5	Synthesis based	IDegLira	Ins	CE
Barnett AH 2018**[27]**	UK	NM	NM	Life	CDM	3.5	3.5	Single study	Lira	DPP4i	CE
Drummond RS 2018**[44]**	UK	58.3	NM	1	Excel sheet	none	none	Single study	IDegLira	Ins	CE
Ericsson A 2018**[67]**[*]	Sweden	59.3	NM	40	SIHECM	3	3	Multi-study	Lira	Ins+GLP	Lira CE
Ishii H 2018**[53]**	Japan	56.83	71	Life	CDM	2	2	Single study	Dula	Ins	CE
Vega-Hernandez G 2017**[87]**	UK	55.3	52.9	Life	CDM	3.5	3.5	Synthesis based	Lira	SGLT	CE
Chakravarty A 2018**[78]**	USA	NM	NM	1	DAM	none	none	Synthesis based	GLP1	SGLT+SU	not CE
Lee WC 2011**[85]**	USA	56.1	49.4	35	IMS	3	3	Single study	Lira	TZD	CE
Chuang LH 2016**[69]**	UK	54.9	61.1	40	Cardiff	3.5	3.5	Synthesis based	Exen	Dula	Exen CE
Chuang LH 2016**[69]**	UK	54.9	61.1	40	Cardiff	3.5	3.5	Synthesis based	Lira	Exen	Exen CE
Chuang LH 2016**[69]**	UK	54.9	61.1	40	Cardiff	3.5	3.5	Synthesis based	Exen	Lixi	Exen CE
Basson M 2018**[88]**	France	56.7	51	Life	CDM	4	4	Synthesis based	Dula	Exen	Dula dominant
Tzanetakos C 2018**[77]**	Greece	57.9	53	Life	Cardiff	3.5	3.5	Multi-study	Exen	Ins+GLP	CE

Suppl.Table 1 Characteristics of selected studies for GLP1 pooling (cont.)

Author	Country	(yr) Age	Men	(yr) TH	Model	DC	DE	Study type	Intervention	Comparison	GLP1 CE
Tzanetakos C 2018**[77]**	Greece	57.9	53	Life	Cardiff	3.5	3.5	Multi-study	Exen	Ins+GLP	CE
Gu S 2017**[63]**	China	50.6	54	40	Cardiff	3	3	Multi-study	Exen	Ins	CE
Hunt B 2017**[52]**	UK	NM	NM	Life	IMS	3.5	3.5	Synthesis based	Lira	Exen	Lira CE
Hunt B 2017**[74]**	UK	NM	NM	Life	IMS	3.5	3.5	Synthesis based	Lira	Lixi	Lira CE

Cardiff, Cardiff diabetes model; CDC, centre for disease control diabetes model; CDM, centre for outcomes research diabetes model; CE, cost-effective; DAM, a short-term decision analytic model; DC, discount rate for cost; DE, discount rate for effectiveness; DES, discrete event simulation model; Dula, dulaglutide; Exen, exenatide; IHE, IHE cohort Model; IMS, IMS core diabetes model; Ins, insulin; Lira, liraglutide; Lixi, lixisenatide; NA, not applicable; NM, not mentioned; SU, sulfonylurea; SIHECM, Swedish institute for health economics cohort model for T2DM; TAM, the archimedes model; TH, time horizon; TZD, thiazolidines; UKPDS, United Kingdom prospective diabetes study outcomes forecast model
*societal perspective

Suppl. Table 2 Estimation of ICER and INB along with variances of individual studies

Author	Status	Threshold	ICER	INB	INB Variance
**DPP4i**					
Sinha A 2010[1]	HIC	56,924	-122,835	-6,058	75,890,095
Davies MJ 2012 [2]	HIC	34,905	-341	3,063	30,570,369
Guillermin AL 2012[3]	HIC	56,206	-8,767	18,452	75,890,095
Lee WC 2012[4]	HIC	54,486	27,286	5,267	75,890,095
Mezquita Raya P 2013[5]	HIC	31,253	-24,800	1,529	36,623,523
Steen Carlsson K 2014[6]	HIC	58,024	76,906	-11,643	4316,649,739
Perez A 2015[7]	HIC	46,880	21,307	12,007	71,890,710
Bruhn D 2016[8]	HIC	51,771	-43,238	3,077	96,823,864
Roussel R 2016[9]	HIC	37,833	-183	6,373	55,403,868
Barnett AH 2018[10]	HIC	29,382	3,427	1,172	24,524,439
**Sulfonylurea**					
Watkins JB 2006[11]	HIC	62,757	40,165	23,848	122,837,107
Minshall ME 2008[12]	HIC	62,757	58,628	9,166	122,837,107
Sinha A 2010[1]	HIC	56,924	3,17,565	-22,285	122,837,107
Davies MJ 2012[2]	HIC	34,905	7,465	5,664	32,946,884
Gao L 2012[13#]	UMIC	33,967	18,745	-13,142	32,946,884
Steen Carlsson K 2014[6]	HIC	58,024	90,550	-18,393	6536,773,623
Roussel R 2016[9]	HIC	37,833	59,464	2,692	58,155,006
**Insulin**					
Watkins JB 2006[11]	HIC	62,757	16,317	61,502	88,454,188
Ray JA 2007[14]	HIC	57,955	49,311	6,421	71,051,822
Woehl A 2008[15*]	HIC	54,241	-52,702	-9,817,555	*
Brandle M 2009[16]	HIC	6,411	3,155	1,731	1,252,907
Mittendorf T 2009[17]	HIC	43,638	16,120	10,207	10,320,030
Beaudet A 2011[18]	HIC	51,239	-608	6,025	65,913,089
Goodall G 2011[19]	HIC	52,359	30,697	15,538	114,625,014
Gaebler JA 2012[20]	HIC	56,206	-29,766	20,237	88,454,188
Samyshkin Y 2012[21]	HIC	56,206	13,776	9,349	88,454,188
Fonseca T 2013[22]	HIC	46,880	85,553	-3,005	7,16,18,226
Kiadaliri AA 2014[23]	HIC	57,999	17,780	10,055	114,625,014
Zhang Y 2014[24]	HIC	52,610	2,32,435	-2,287	88,454,188
Deng J 2015[25#]	UMIC	12,988	-13,417	50,027	225,494,973
Huetson P 2015[26]	HIC	65,072	8,904	5,518	108,749,867
Bruhn D 2016[8]	HIC	51,771	22,492	674	83,559,689
Davies MJ 2016[27]	HIC	44,356	-17,720	5,602	8,355,829
Gordon J 2016[28]	HIC	58,156	229	37,072	58,259,834
Ericsson A 2017[29#]	HIC	34,877	657	56,161	58,259,834
Hunt B 2017[30]	HIC	25,489	-13,961	17,367	58,259,834
Hunt B 2017[31]	HIC	1,03,419	1,30,232	10,468	343,975,971
Kvapil M 2017[32#]	HIC	83,957	29,845	17,472	181,158,025

Lasalvia P 2017[33#]	UMIC	15	15	0	484
Psota M 2017[34#]	HIC	42,049	21,800	6,628	28,498,717
Drummond RS 2018[35]	HIC	29,382	8,703	1,059	8,355,829
Ishii H 2018[36]	HIC	49,844	1,423	14,631	475,568,935
Tzanetakos C 2018[37#]	HIC	61,183	7,747	24,557	8,775,698
Gu S 2017[38#]	UMIC	13,980	-19,310	63,393	334,276,472
**Insulin plus SU**					
Ivanova A 2011[39]	UMIC	50,282	51,412	-272	419,525,884
Zhang Y 2014[24]	HIC	52,610	-8,71,813	-15,431	419,525,884
**Insulin plus DPP4i**					
Kiadaliri AA 2014[23]	HIC	57,999	37,028	2,097	88,921,118
Tzanetakos C 2014[40]	HIC	55,573	-9,45,378	5,755	88,921,118
**Insulin plus GLP1**					
Ericsson A 2017[29#]	HIC	34,877	4,977	14,358	192,144,724
Hunt B 2017[41]	HIC	1,03,419	-26,03,362	20,638	419,525,884
Kvapil M 2017[32#]	HIC	83,957	-9,954	2,202	243,085,724
Lasalvia P 2017[33#]	UMIC	15	-0	1	419,525,884
Ericsson A 2018[42]	HIC	58,156	4,888	41,072	192,144,724
**Lira versus Lixi**					
Mezquita Raya P 2013[5]	HIC	31,021	-20,858	3,466	36,302,276
Hunt B 2017[43]	HIC	40,834	2,750	4,570	83,143
Hunt B 2017[44]	HIC	29,571	6,386	2,060	24,111,296
Mezquita-Raya P 2017[45]	HIC	31,021	-9,698	3,585	36,952,568
Hunt B 2017[32]	HIC	44,356	3,745	3,505	57,040,382
**Lira versus Exen**					
Lee WC 2010[46]	HIC	56,206	49,482	3,072	93,021,094
Petrova G 2011[47]	UMIC	85,847	26,783	5,983	115,625,622
Valentine W 2011[48]	HIC	45,728	6,324	6,031	57,365,156
Valentine W 2011[48]	HIC	9,042	-7,915	-480	5,476,184
Valentine W 2011[48]	HIC	8,336	15,223	-988	3,766,955
Valentine W 2011[48]	HIC	72,668	12,173	10,165	156,246,556
Valentine W 2011[48]	HIC	85,561	16,747	12,256	263,436,969
Valentine W 2011[48]	HIC	83,325	25,567	9,483	162,201,874
Zhang X 2016[49]	UMIC	14,622	4,119	3,939	39,894,261
Chuang LH 2016[50]	HIC	44,378	-33,830	1,741	7,903,929
Tzanetakos C 2018[37#]	HIC	61,183	-3,095	2,193	5,460,098
Hunt B 2017[51]	HIC	44,356	11,924	957	56,913,980
**IDegLira versus Insulin**					
Davies MJ 2016[27]	HIC	44,356	-17,720	5,602	8,355,829
Ericsson A 2017[29#]	HIC	34,877	657	56,161	58,259,834
Hunt B 2017[41]	HIC	25,489	-13,961	17,367	58,259,834
Hunt B 2017[31]	HIC	1,03,419	1,30,232	10,468	343,975,971

Kvapil M 2017[32#]	HIC	83,957	29,845	17,472	181,158,025
Psota M 2017[34#]	HIC	42,049	21,800	6,628	28,498,717
Drummond RS 2018[35]	HIC	29,382	8,703	1,059	8,355,829
**Thiazolidines**					
Watkins JB 2006[11]	HIC	62,757	-1,046	76,564	88,579,917
Gaebler JA 2012[20]	HIC	56,206	-26,564	21,520	88,579,917
Guillermin AL 2012[3]	HIC	56,206	-4,298	14,763	82,907,146

* As the study has huge INB and no other similar study its variance was not considered from other studies.
# Countries with GDP based thresholds

References:

1. Sinha A, Rajan M, Hoerger T, Pogach L. Costs and consequences associated with newer medications for glycemic control in type 2 diabetes. *Diabetes Care.* 2010;33(4):695-700.
2. Davies MJ, Chubb BD, Smith IC, Valentine WJ. Cost-utility analysis of liraglutide compared with sulphonylurea or sitagliptin, all as add-on to metformin monotherapy in Type 2 diabetes mellitus. *Diabet Med.* 2012;29(3):313-320.
3. Guillermin AL, Lloyd A, Best JH, DeYoung MB, Samyshkin Y, Gaebler JA. Long-term cost-consequence analysis of exenatide once weekly vs sitagliptin or pioglitazone for the treatment of type 2 diabetes patients in the United States. *J Med Econ.* 2012;15(4):654-663.
4. Lee WC, Samyshkin Y, Langer J, Palmer JL. Long-term clinical and economic outcomes associated with liraglutide versus sitagliptin therapy when added to metformin in the treatment of type 2 diabetes: a CORE Diabetes Model analysis. *J Med Econ.* 2012;15 Suppl 2:28-37.
5. Mezquita Raya P, Perez A, Ramirez de Arellano A, Briones T, Hunt B, Valentine WJ. Incretin therapy for type 2 diabetes in Spain: a cost-effectiveness analysis of liraglutide versus sitagliptin. *Diabetes Ther.* 2013;4(2):417-430.
6. Steen Carlsson K, Persson U. Cost-effectiveness of add-on treatments to metformin in a Swedish setting: liraglutide vs sulphonylurea or sitagplitin. *J Med Econ.* 2014;17(9):658-669.
7. Perez A, Mezquita Raya P, Ramirez de Arellano A, Briones T, Hunt B, Valentine WJ. Cost-Effectiveness Analysis of Incretin Therapy for Type 2 Diabetes in Spain: 1.8 mg Liraglutide Versus Sitagliptin. *Diabetes Ther.* 2015;6(1):61-74.
8. Bruhn D, Martin AA, Tavares R, Hunt B, Pollock RF. Cost-utility of albiglutide versus insulin lispro, insulin glargine, and sitagliptin for the treatment of type 2 diabetes in the US. *J Med Econ.* 2016;19(7):672-683.
9. Roussel R, Martinez L, Vandebrouck T, et al. Evaluation of the long-term cost-effectiveness of liraglutide therapy for patients with type 2 diabetes in France. *J Med Econ.* 2016;19(2):121-134.
10. Barnett AH, Arnoldini S, Hunt B, Subramanian G, Hoxer CS. Switching from sitagliptin to liraglutide to manage patients with type 2 diabetes in the UK: A long-term cost-effectiveness analysis. *Diabetes Obes Metab.* 2018;20(8):1921-1927.
11. Watkins JB, Minshall ME, Sullivan SD. Application of economic analyses in U.S. managed care formulary decisions: a private payer's experience. *J Manag Care Pharm.* 2006;12(9):726-735.
12. Minshall ME, Oglesby AK, Wintle ME, Valentine WJ, Roze S, Palmer AJ. Estimating the long-term cost-effectiveness of exenatide in the United States: an adjunctive treatment for type 2 diabetes mellitus (Structured abstract). *Value in Health.* 2008;11(1):22-33.
13. Gao L, Zhao FL, Li SC. Cost-utility analysis of liraglutide versus glimepiride as add-on to metformin in type 2 diabetes patients in China. *Int J Technol Assess Health Care.* 2012;28(4):436-444.
14. Ray JA, Boye KS, Yurgin N, et al. Exenatide versus insulin glargine in patients with type 2 diabetes in the UK: a model of long-term clinical and cost outcomes. *Curr Med Res Opin.* 2007;23(3):609-622.
15. Woehl A, Evans M, Tetlow AP, McEwan P. Evaluation of the cost effectiveness of exenatide versus insulin glargine in patients with sub-optimally controlled type 2 diabetes in the United Kingdom. *Cardiovasc Diabetol.* 2008;7:24.
16. Brandle M, Erny-Albrecht KM, Goodall G, Spinas GA, Streit P, Valentine WJ. Exenatide versus insulin glargine: a cost-effectiveness evaluation in patients with Type 2 diabetes in Switzerland. *Int J Clin Pharmacol Ther.* 2009;47(8):501-515.

17. Mittendorf T, Smith-Palmer J, Timlin L, Happich M, Goodall G. Evaluation of exenatide vs. insulin glargine in type 2 diabetes: cost-effectiveness analysis in the German setting. *Diabetes Obes Metab.* 2009;11(11):1068-1079.
18. Beaudet A, Palmer JL, Timlin L, et al. Cost-utility of exenatide once weekly compared with insulin glargine in patients with type 2 diabetes in the UK. *J Med Econ.* 2011;14(3):357-366.
19. Goodall G, Costi M, Timlin L, et al. [Cost-effectiveness of exenatide versus insulin glargine in Spanish patients with obesity and type 2 diabetes mellitus]. *Endocrinol Nutr.* 2011;58(7):331-340.
20. Gaebler JA, Soto-Campos G, Alperin P, et al. Health and economic outcomes for exenatide once weekly, insulin, and pioglitazone therapies in the treatment of type 2 diabetes: a simulation analysis. *Vasc Health Risk Manag.* 2012;8:255-264.
21. Samyshkin Y, Guillermin AL, Best JH, Brunell SC, Lloyd A. Long-term cost-utility analysis of exenatide once weekly versus insulin glargine for the treatment of type 2 diabetes patients in the US. *J Med Econ.* 2012;15 Suppl 2:6-13.
22. Fonseca T, Clegg J, Caputo G, Norrbacka K, Dilla T, Alvarez M. The cost-effectiveness of exenatide once weekly compared with exenatide twice daily and insulin glargine for the treatment of patients with type two diabetes and body mass index >/=30 kg/m(2) in Spain. *J Med Econ.* 2013;16(7):926-938.
23. Kiadaliri AA, Gerdtham UG, Eliasson B, Carlsson KS. Cost-utility analysis of glucagon-like Peptide-1 agonists compared with dipeptidyl peptidase-4 inhibitors or neutral protamine hagedorn Basal insulin as add-on to metformin in type 2 diabetes in sweden. *Diabetes Ther.* 2014;5(2):591-607.
24. Zhang Y, McCoy RG, Mason JE, Smith SA, Shah ND, Denton BT. Second-line agents for glycemic control for type 2 diabetes: are newer agents better? *Diabetes Care.* 2014;37(5):1338-1345.
25. Deng J, Gu S, Shao H, Dong H, Zou D, Shi L. Cost-effectiveness analysis of exenatide twice daily (BID) vs insulin glargine once daily (QD) as add-on therapy in Chinese patients with Type 2 diabetes mellitus inadequately controlled by oral therapies. *J Med Econ.* 2015;18(11):974-989.
26. Huetson P, Palmer JL, Levorsen A, Fournier M, Germe M, McLeod E. Cost-effectiveness of once daily GLP-1 receptor agonist lixisenatide compared to bolus insulin both in combination with basal insulin for the treatment of patients with type 2 diabetes in Norway. *J Med Econ.* 2015;18(8):573-585.
27. Davies MJ, Glah D, Chubb B, Konidaris G, McEwan P. Cost Effectiveness of IDegLira vs. Alternative Basal Insulin Intensification Therapies in Patients with Type 2 Diabetes Mellitus Uncontrolled on Basal Insulin in a UK Setting. *Pharmacoeconomics.* 2016;34(9):953-966.
28. Gordon J, McEwan P, Sabale U, Kartman B, Wolffenbuttel BH. The cost-effectiveness of exenatide twice daily (BID) vs insulin lispro three times daily (TID) as add-on therapy to titrated insulin glargine in patients with type 2 diabetes. *J Med Econ.* 2016;19(12):1167-1174.
29. Ericsson A, Lundqvist A. Cost Effectiveness of Insulin Degludec Plus Liraglutide (IDegLira) in a Fixed Combination for Uncontrolled Type 2 Diabetes Mellitus in Sweden. *Appl Health Econ Health Policy.* 2017;15(2):237-248.
30. Hunt B, Glah D, van der Vliet M. Modeling the Long-Term Cost-Effectiveness of IDegLira in Patients with Type 2 Diabetes Who are Failing To Meet Glycemic Targets on Basal Insulin Alone in The Netherlands. *Diabetes Ther.* 2017;8(4):753-765.
31. Hunt B, Mocarski M, Valentine WJ, Langer J. IDegLira Versus Insulin Glargine U100: A Long-term Cost-effectiveness Analysis in the US Setting. *Diabetes Ther.* 2017;8(3):531-544.
32. Kvapil M, Prazny M, Holik P, Rychna K, Hunt B. Cost-Effectiveness of IDegLira Versus Insulin Intensification Regimens for the Treatment of Adults with Type 2 Diabetes in the Czech Republic. *Diabetes Ther.* 2017;8(6):1331-1347.
33. Lasalvia P, Baquero L, Otalora-Esteban M, Castaneda-Cardona C, Rosselli D. Cost Effectiveness of Dulaglutide Compared with Liraglutide and Glargine in Type 2 Diabetes Mellitus Patients in Colombia. *Value Health Reg Issues.* 2017;14:35-40.
34. Psota M, Psenkova MB, Racekova N, Ramirez de Arellano A, Vandebrouck T, Hunt B. Cost-effectiveness analysis of IDegLira versus basal-bolus insulin for patients with type 2 diabetes in the Slovak health system. *Clinicoecon Outcomes Res.* 2017;9:749-762.
35. Drummond R, Malkin S, Du Preez M, Lee XY, Hunt B. The management of type 2 diabetes with fixed-ratio combination insulin degludec/liraglutide (IDegLira) versus basal-bolus therapy (insulin glargine U100 plus insulin aspart): A short-term cost-effectiveness analysis in the UK setting. *Diabetes Obes Metab.* 2018;20(10):2371-2378.
36. Ishii H, Madin-Warburton M, Strizek A, Thornton-Jones L, Suzuki S. The cost-effectiveness of dulaglutide versus insulin glargine for the treatment of type 2 diabetes mellitus in Japan. *J Med Econ.* 2018;21(5):488-496.
37. Tzanetakos C, Bargiota A, Kourlaba G, Gourzoulidis G, Maniadakis N. Cost Effectiveness of Exenatide Once Weekly Versus Insulin Glargine and Liraglutide for the Treatment of Type 2 Diabetes Mellitus in Greece. *Clin Drug Investig.* 2018;38(1):67-77.

38. Gu S, Wang X, Qiao Q, Gao W, Wang J, Dong H. Cost-effectiveness of exenatide twice daily vs insulin glargine as add-on therapy to oral antidiabetic agents in patients with type 2 diabetes in China. *Diabetes Obes Metab.* 2017;19(12):1688-1697.
39. Ivanova A, Petrova G, Wrona W, Valov V, Stoimenova A, Czech M. Modelling the Long-Term Effect of Diabetes Therapy. An Example with Liraglutide. *Comptes Rendus De L Academie Bulgare Des Sciences.* 2011;64(3):449-456.
40. Tzanetakos C, Melidonis A, Verras C, Kourlaba G, Maniadakis N. Cost-effectiveness analysis of liraglutide versus sitagliptin or exenatide in patients with inadequately controlled Type 2 diabetes on oral antidiabetic drugs in Greece. *BMC Health Serv Res.* 2014;14:419.
41. Hunt B, Mocarski M, Valentine WJ, Langer J. Evaluation of the long-term cost-effectiveness of IDegLira versus liraglutide added to basal insulin for patients with type 2 diabetes failing to achieve glycemic control on basal insulin in the USA. *J Med Econ.* 2017;20(7):663-670.
42. Ericsson A, Glah D, Lorenzi M, Jansen JP, Fridhammar A. Cost-effectiveness of liraglutide versus lixisenatide as add-on therapies to basal insulin in type 2 diabetes. *PLoS One.* 2018;13(2):e0191953.
43. Hunt B, Kragh N, McConnachie CC, Valentine WJ, Rossi MC, Montagnoli R. Long-term Cost-effectiveness of Two GLP-1 Receptor Agonists for the Treatment of Type 2 Diabetes Mellitus in the Italian Setting: Liraglutide Versus Lixisenatide. *Clin Ther.* 2017;39(7):1347-1359.
44. Hunt B, Vega-Hernandez G, Valentine WJ, Kragh N. Evaluation of the long-term cost-effectiveness of liraglutide vs lixisenatide for treatment of type 2 diabetes mellitus in the UK setting. *Diabetes Obes Metab.* 2017;19(6):842-849.
45. Mezquita-Raya P, Ramirez de Arellano A, Kragh N, et al. Liraglutide Versus Lixisenatide: Long-Term Cost-Effectiveness of GLP-1 Receptor Agonist Therapy for the Treatment of Type 2 Diabetes in Spain. *Diabetes Ther.* 2017;8(2):401-415.
46. Lee WC, Conner C, Hammer M. Results of a model analysis of the cost-effectiveness of liraglutide versus exenatide added to metformin, glimepiride, or both for the treatment of type 2 diabetes in the United States. *Clin Ther.* 2010;32(10):1756-1767.
47. Petrova G, Ivanova A, Czech M, et al. Liraglutide vs. exenatide in combination with metformin and/or sulfonylurea therapy in type 2 diabetes mellitus therapy in Bulgaria: A modelling study. *Comptes Rendus de L'Academie Bulgare des Sciences.* 2011;64(10):1507-1514.
48. Valentine WJ, Palmer AJ, Lammert M, Langer J, Brandle M. Evaluating the long-term cost-effectiveness of liraglutide versus exenatide BID in patients with type 2 diabetes who fail to improve with oral antidiabetic agents. *Clin Ther.* 2011;33(11):1698-1712.
49. Zhang X, Liu S, Li Y, Wang Y, Tian M, Liu G. Long-Term Effectiveness and Cost-Effectiveness of Metformin Combined with Liraglutide or Exenatide for Type 2 Diabetes Mellitus Based on the CORE Diabetes Model Study. *PLoS One.* 2016;11(6):e0156393.
50. Chuang LH, Verheggen BG, Charokopou M, Gibson D, Grandy S, Kartman B. Cost-effectiveness analysis of exenatide once-weekly versus dulaglutide, liraglutide, and lixisenatide for the treatment of type 2 diabetes mellitus: an analysis from the UK NHS perspective. *J Med Econ.* 2016;19(12):1127-1134.
51. Hunt B, Ye Q, Valentine WJ, Ashley D. Evaluating the Long-Term Cost-Effectiveness of Daily Administered GLP-1 Receptor Agonists for the Treatment of Type 2 Diabetes in the United Kingdom. *Diabetes Ther.* 2017;8(1):129-147.
