# Supplementary material for: Glucagon-like peptide 1 agonists for treatment of patients with type 2 diabetes who fail metformin monotherapy: systematic review and meta-analysis of economic evaluation studies
Source: BMJ Open Diabetes Res Care. 2020 Jul 19;8(1):e001020. doi: 10.1136/bmjdrc-2019-001020 (PMC7371226; doi:10.1136/bmjdrc-2019-001020)
Supplement: Supplementary data [file bmjdrc-2019-001020supp004.pdf]

	PART A. Overall checklist for bias in economic evaluation											PART B. Model-specific aspects of bias in economic evaluation, I Bias related to structure				PART B. Model-specific aspects of bias in economic evaluation, II Bias related to data						III Bias related to consistency
	Narrow perspective bias	Inefficient comparator bias*	Cost measurement omission bias	Intermittent data collection bias	Invalid valuation bias	Ordinal ICER bias	Double-counting bias	Inappropriate discounting bias	Limited sensitivity analysis bias§	Sponsor bias	Reporting and dissemination bias	Structural assumptions bias	No treatment comparator bias*	Wrong model bias	Limited time horizon bias	Bias related to data identification	Bias related to baseline data	Bias related to treatment effects	Bias related to quality-of-life weights (utilities)	Non-transparent data incorporation bias	Limited scope bias§	Bias related to internal consistency
**Watkins, J. B**	partly	yes	partly	yes	partly	yes	unclear	no	no	yes	unclear	yes	yes	yes	partly	partly	partly	yes	no	partly	unclear	unclear
**Ishii, H**	yes	yes	yes	yes	yes	yes	partly	yes	yes	yes	unclear	yes	yes	yes	yes	yes	yes	yes	yes	yes	yes	unclear
**Beaudet, A**	yes	yes	yes	yes	yes	yes	yes	yes	yes	yes	unclear	yes	yes	yes	yes	yes	yes	yes	yes	yes	yes	unclear
**Woehl, A**	yes	yes	yes	yes	yes	yes	partly	yes	unclear	partly	unclear	partly	yes	yes	partly	yes	yes	yes	yes	yes	partly	unclear
**Ray, J. A**	partly	yes	yes	yes	yes	yes	unclear	yes	partly	partly	unclear	yes	yes	yes	partly	yes	yes	yes	yes	yes	partly	unclear
**Brandle, M**	yes	yes	yes	yes	yes	yes	unclear	yes	yes	partly	unclear	yes	yes	yes	yes	yes	yes	yes	yes	yes	unclear	unclear
**Gaebler, J. A**	yes	yes	yes	yes	yes	yes	partly	yes	yes	yes	unclear	yes	yes	yes	yes	yes	yes	yes	yes	yes	yes	unclear
**Hunt, B**	yes	yes	yes	yes	yes	yes	partly	yes	yes	yes	unclear	yes	yes	yes	yes	yes	yes	yes	yes	yes	yes	unclear
**Mezquita Raya, P**	yes	yes	yes	yes	yes	yes	partly	yes	yes	yes	unclear	yes	yes	yes	yes	yes	yes	yes	yes	yes	yes	unclear
**Mezquita-Raya, P**	yes	yes	yes	yes	yes	yes	partly	yes	yes	yes	unclear	yes	yes	yes	yes	yes	yes	yes	yes	yes	yes	unclear
**Petrova, G**	yes	yes	partly	yes	partly	yes	partly	yes	yes	no	unclear	yes	yes	yes	yes	yes	yes	yes	yes	yes	yes	unclear
**Lee, W. C**	yes	yes	yes	yes	yes	yes	partly	yes	yes	yes	unclear	yes	yes	yes	yes	yes	yes	yes	yes	yes	yes	unclear
**Hunt, B**	yes	yes	yes	yes	yes	yes	partly	yes	yes	no	unclear	yes	yes	yes	yes	yes	yes	yes	yes	yes	yes	unclear
**Samyshkin, Y**	yes	yes	yes	yes	yes	yes	partly	yes	yes	yes	unclear	yes	yes	yes	yes	yes	yes	yes	yes	yes	yes	unclear
**Zhang, X**	yes	yes	yes	yes	partly	yes	partly	yes	yes	yes	unclear	yes	yes	yes	yes	yes	yes	partly	yes	yes	yes	unclear
**Drummond, R. S**	yes	yes	partly	yes	partly	yes	partly	NA	yes	yes	unclear	unclear	yes	unclear	no	yes	partly	yes	partly	yes	partly	unclear
**Ivanova, A**	partly	unclear	unclear	yes	partly	yes	partly	unclear	yes	no	unclear	yes	partly	yes	partly	partly	partly	partly	partly	partly	unclear	unclear
**Zhang, Y**	no	yes	partly	unclear	yes	yes	partly	yes	partly	yes	unclear	unclear	yes	unclear	unclear	yes	yes	yes	yes	partly	partly	unclear
**Barnett, A. H**	yes	yes	yes	yes	yes	yes	partly	yes	yes	yes	unclear	yes	yes	yes	yes	yes	yes	yes	yes	yes	yes	unclear
**Davies, M. J**	yes	yes	yes	yes	yes	yes	partly	yes	yes	yes	unclear	yes	yes	yes	yes	yes	yes	yes	yes	yes	yes	unclear
**Ericsson, A.**	yes	yes	yes	yes	partly	yes	partly	yes	partly	yes	unclear	yes	yes	yes	yes	yes	yes	yes	yes	partly	partly	unclear
**Psota, M**	yes	yes	yes	yes	yes	yes	partly	yes	yes	yes	unclear	yes	yes	yes	yes	yes	yes	yes	yes	yes	yes	unclear
**Perez, A**	yes	yes	yes	yes	yes	yes	partly	yes	partly	yes	unclear	yes	yes	yes	yes	yes	yes	yes	yes	yes	partly	unclear
**Tzanetakos, C**	yes	yes	yes	yes	yes	yes	partly	yes	partly	yes	unclear	yes	yes	yes	yes	yes	yes	yes	yes	yes	yes	unclear
**Steen Carlsson, K.**	yes	yes	yes	yes	yes	yes	partly	yes	yes	yes	unclear	yes	yes	yes	yes	yes	yes	yes	yes	yes	yes	unclear
**Gordon, J**	yes	yes	yes	yes	yes	yes	partly	yes	yes	yes	unclear	yes	yes	yes	yes	yes	yes	yes	yes	yes	yes	unclear
**Kvapil, M**	yes	yes	yes	yes	yes	yes	partly	yes	yes	yes	unclear	yes	yes	yes	yes	yes	yes	yes	yes	yes	yes	unclear
**Sinha, A**	yes	yes	yes	yes	yes	yes	partly	yes	partly	yes	unclear	yes	yes	yes	yes	yes	yes	yes	yes	yes	unclear	unclear
**Gao, L**	yes	yes	yes	yes	yes	yes	partly	yes	yes	yes	unclear	yes	yes	yes	yes	yes	yes	yes	yes	yes	partly	unclear
**Roussel, R**	yes	yes	yes	yes	yes	yes	partly	yes	yes	yes	unclear	yes	yes	yes	yes	yes	yes	yes	yes	yes	yes	unclear
**Huetson, P**	yes	yes	yes	yes	yes	yes	partly	yes	yes	yes	unclear	yes	yes	yes	yes	yes	yes	yes	yes	yes	yes	unclear
**Kiadaliri, A. A**	yes	yes	yes	yes	yes	yes	partly	yes	yes	yes	unclear	yes	yes	yes	yes	yes	yes	yes	yes	yes	yes	unclear
**Davies, M. J**	yes	yes	yes	yes	yes	yes	partly	yes	yes	yes	unclear	yes	yes	yes	yes	yes	yes	yes	yes	yes	yes	unclear
**Mittendorf, T**	yes	yes	yes	yes	yes	yes	partly	yes	yes	yes	unclear	yes	yes	yes	yes	yes	yes	yes	yes	yes	yes	unclear
**Hunt, B**	yes	yes	yes	yes	yes	yes	partly	yes	yes	yes	unclear	yes	yes	yes	partly	yes	yes	yes	yes	yes	yes	unclear
**Guillermin, A. L**	yes	yes	yes	yes	yes	yes	partly	yes	partly	yes	unclear	yes	yes	yes	yes	yes	yes	yes	yes	yes	partly	unclear
**Hunt, B**	yes	yes	yes	yes	yes	yes	partly	yes	partly	yes	unclear	yes	yes	yes	yes	yes	yes	yes	yes	yes	yes	unclear
**Lasalvia, P**	yes	yes	yes	yes	yes	yes	partly	yes	yes	yes	unclear	yes	yes	yes	no	yes	yes	yes	yes	yes	yes	unclear
**Dilla, T**	yes	yes	yes	yes	yes	yes	partly	yes	yes	yes	unclear	yes	yes	yes	yes	yes	yes	yes	yes	yes	yes	unclear
**Fonseca, T**	yes	yes	yes	yes	yes	yes	partly	yes	yes	yes	unclear	yes	yes	yes	yes	yes	yes	yes	yes	yes	yes	unclear
**Ericsson, A**	yes	yes	yes	yes	yes	yes	partly	yes	yes	yes	unclear	yes	yes	yes	yes	yes	yes	yes	yes	yes	yes	unclear
**Hunt, B**	yes	yes	partly	yes	yes	no	no	yes	partly	yes	unclear	unclear	yes	yes	partly	yes	yes	yes	no	yes	partly	unclear
**Lee, W. C**	yes	yes	yes	yes	yes	yes	partly	yes	yes	partly	unclear	yes	yes	yes	yes	yes	yes	yes	yes	yes	unclear	unclear
**Bruhn, D**	yes	yes	yes	yes	yes	yes	partly	unclear	yes	yes	unclear	yes	yes	yes	yes	yes	yes	yes	yes	yes	yes	unclear
**Deng, J**	yes	yes	yes	yes	yes	yes	partly	yes	yes	yes	unclear	yes	yes	yes	yes	yes	yes	yes	yes	yes	yes	unclear
**Goodall, G**	yes	yes	yes	yes	yes	yes	partly	yes	yes	yes	unclear	yes	yes	yes	yes	yes	yes	yes	yes	yes	yes	unclear
**Lee, W**	yes	yes	yes	yes	yes	yes	partly	yes	yes	yes	unclear	yes	yes	yes	yes	yes	yes	yes	yes	yes	partly	unclear
**Minshall, M. E**	yes	yes	yes	yes	yes	yes	partly	yes	yes	partly	unclear	yes	yes	yes	yes	yes	yes	yes	yes	yes	yes	unclear
**Valentine, W**	yes	yes	yes	yes	yes	yes	partly	yes	yes	yes	yes	yes	yes	yes	yes	yes	yes	yes	yes	yes	yes	unclear
**Hunt, B**	yes	yes	yes	yes	yes	yes	partly	yes	yes	yes	unclear	yes	yes	yes	yes	yes	yes	yes	yes	yes	yes	unclear
**Evans, M**	yes	yes	yes	yes	yes	yes	partly	yes	yes	yes	unclear	yes	yes	yes	yes	yes	yes	yes	yes	yes	yes	unclear
**Lee, W. C**	yes	yes	yes	yes	yes	yes	partly	yes	yes	yes	unclear	yes	yes	yes	yes	yes	yes	yes	yes	yes	yes	unclear
**Vega-Hernandez, G**	yes	yes	yes	yes	yes	yes	partly	yes	yes	yes	unclear	yes	yes	yes	yes	yes	yes	yes	yes	yes	yes	unclear
**Chakravarty, A**	partly	yes	yes	yes	yes	yes	partly	yes	yes	yes	unclear	yes	yes	yes	unclear	yes	yes	yes	yes	yes	partly	unclear
**Basson, M**	yes	yes	yes	yes	yes	yes	partly	yes	yes	yes	unclear	yes	yes	yes	yes	yes	yes	yes	yes	yes	yes	unclear
**Chuang, L. H**	yes	yes	yes	yes	yes	yes	partly	yes	yes	yes	unclear	yes	yes	yes	yes	yes	yes	yes	yes	yes	yes	unclear
**Gu, S**	yes	yes	yes	yes	yes	yes	partly	yes	yes	yes	unclear	yes	yes	yes	yes	yes	yes	yes	yes	yes	partly	unclear
**Hunt, B**	yes	yes	yes	yes	yes	yes	partly	yes	yes	yes	unclear	yes	yes	yes	yes	yes	yes	yes	yes	yes	yes	unclear
**Tzanetakos, C**	yes	yes	yes	yes	yes	yes	partly	yes	yes	yes	unclear	yes	yes	yes	yes	yes	yes	yes	yes	yes	yes	unclear

Suppl. Fig. 1 Risk of bias assessment of GLP1 studies using ECOBIAS checklist

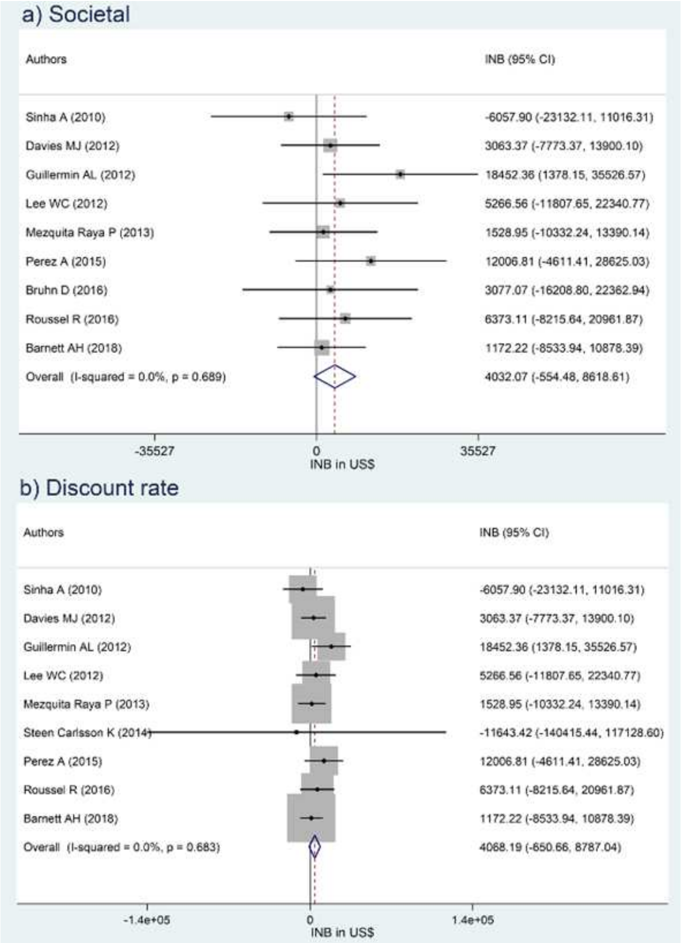

Suppl. Fig. 2 Sensitivity analysis of INB of GLP1 versus DPP4i by omitting the study with a) societal perspective b) no discounting

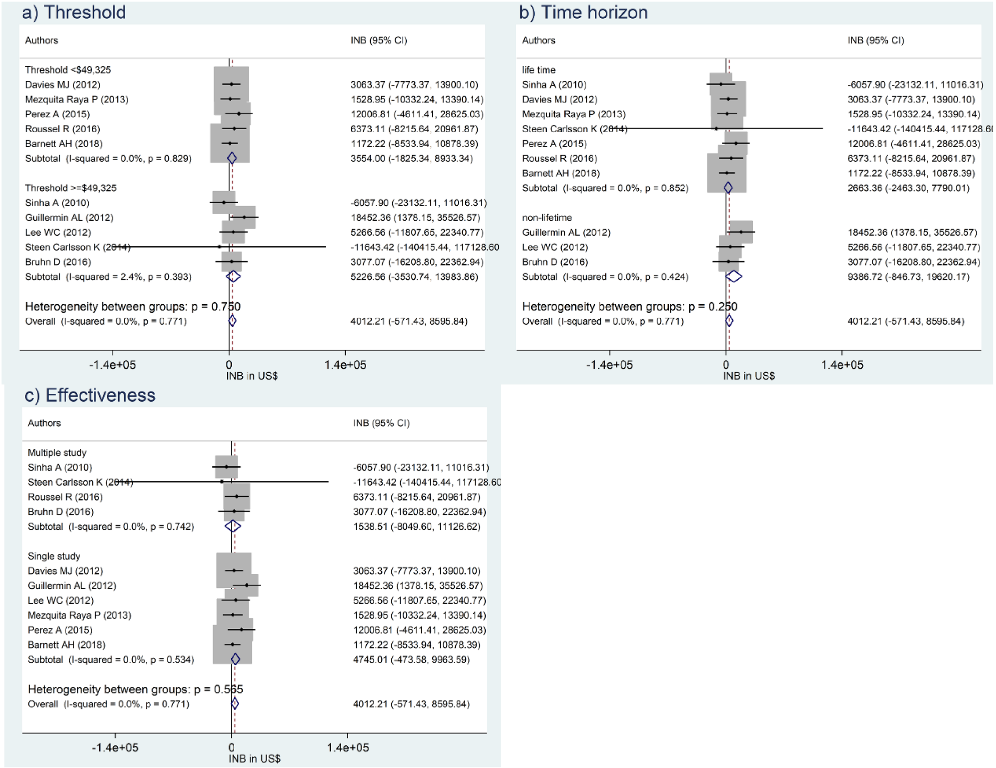

Suppl. Fig. 3 Subgroup analysis of INB of GLP1 versus DPP4i by a) threshold b) time horizon c) effectiveness measure

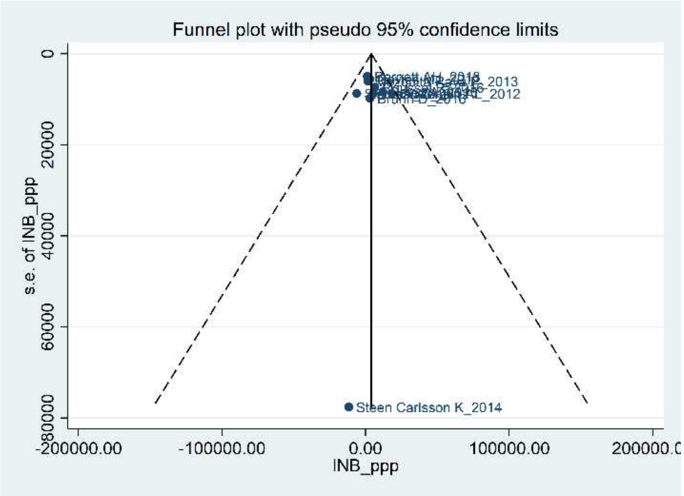

Suppl. Fig. 4 Funnel plot of GLP1 versus DPP4i

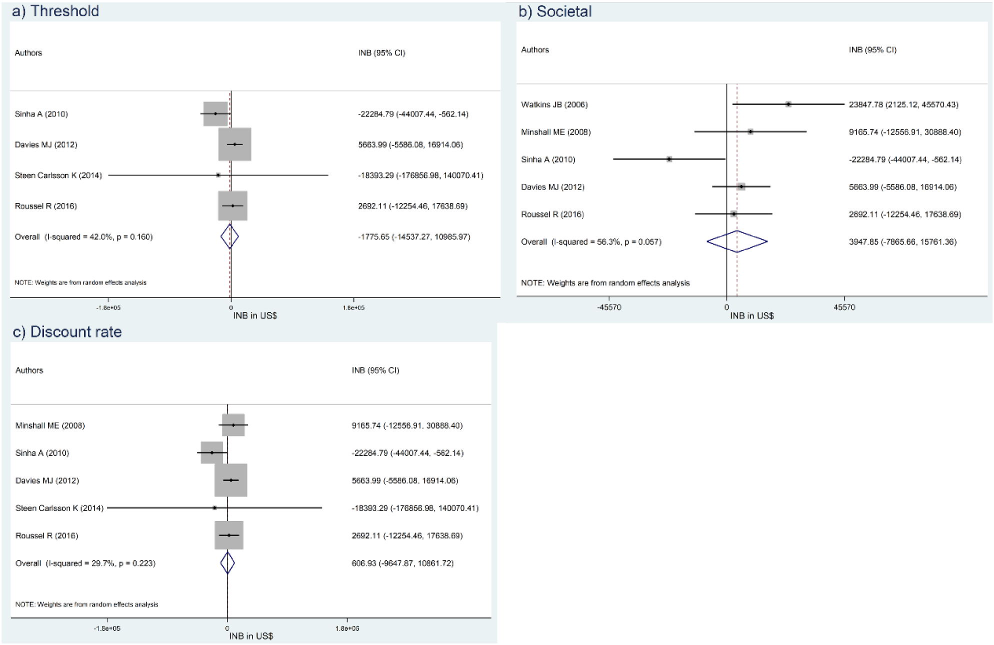

Suppl. Fig. 5 Sensitivity analysis of INB of GLP1 versus Sulfonylurea by omitting the study with a) high threshold b) societal perspective c) no discounting

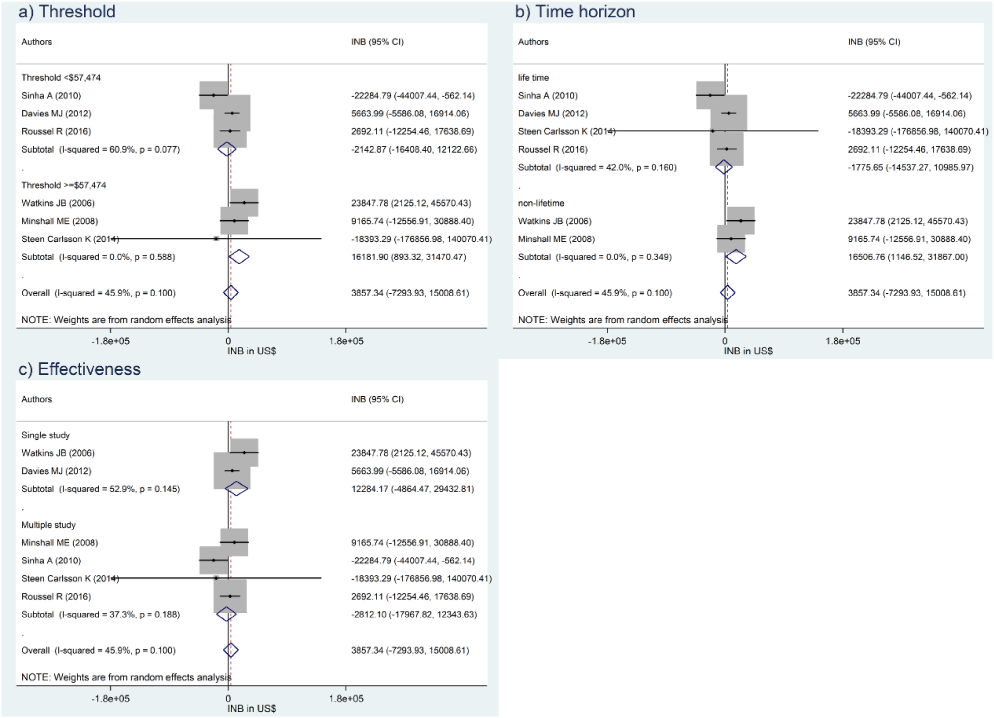

Suppl. Fig. 6 Sub-group analysis of INB of GLP1 versus Sulfonylurea by a) threshold b) time horizon c) effectiveness measure

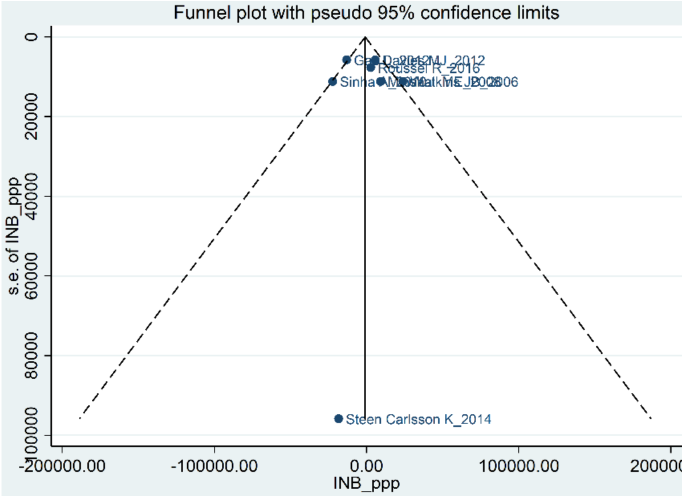

Suppl. Fig. 7 Funnel plot of GLP1 versus Sulfonylurea

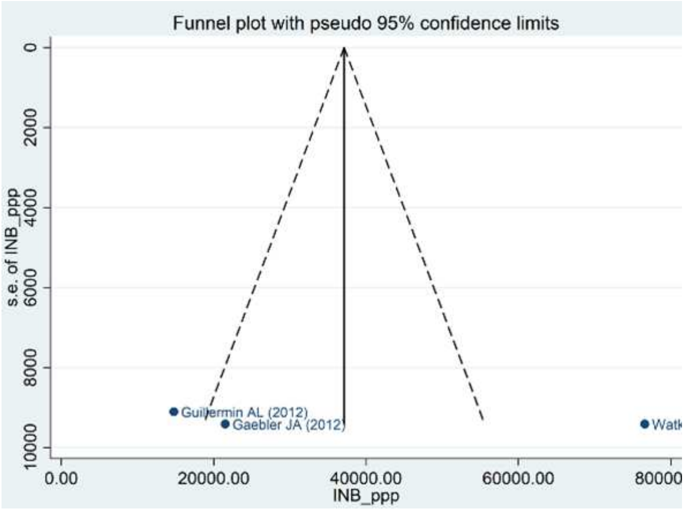

Suppl. Fig. 8 Funnel plot of GLP1 versus Thiazolidines

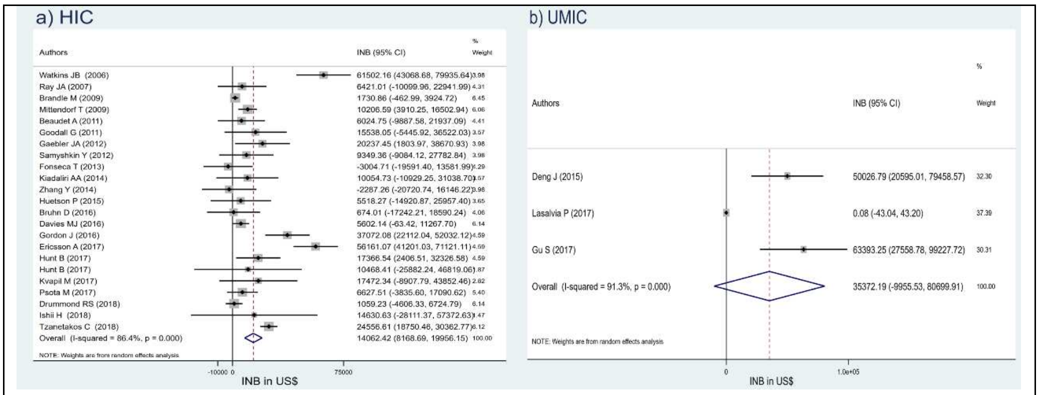

Suppl. Fig. 9 Pooling INB of GLP1 versus Insulin from a) HIC b) UMIC

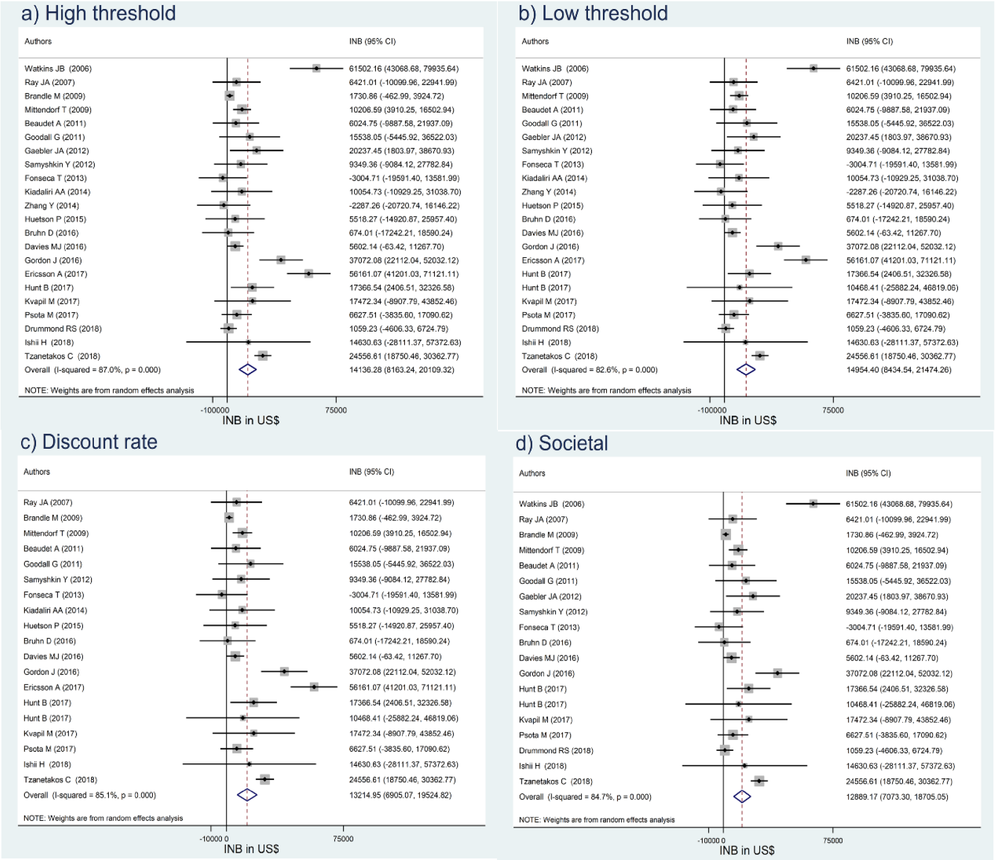

Suppl. Fig. 10 Sensitivity analysis of INB, GLP1 versus Insulin among high-income countries by omitting the studies with a) high threshold b) low threshold c) no discounting d) no societal perspective

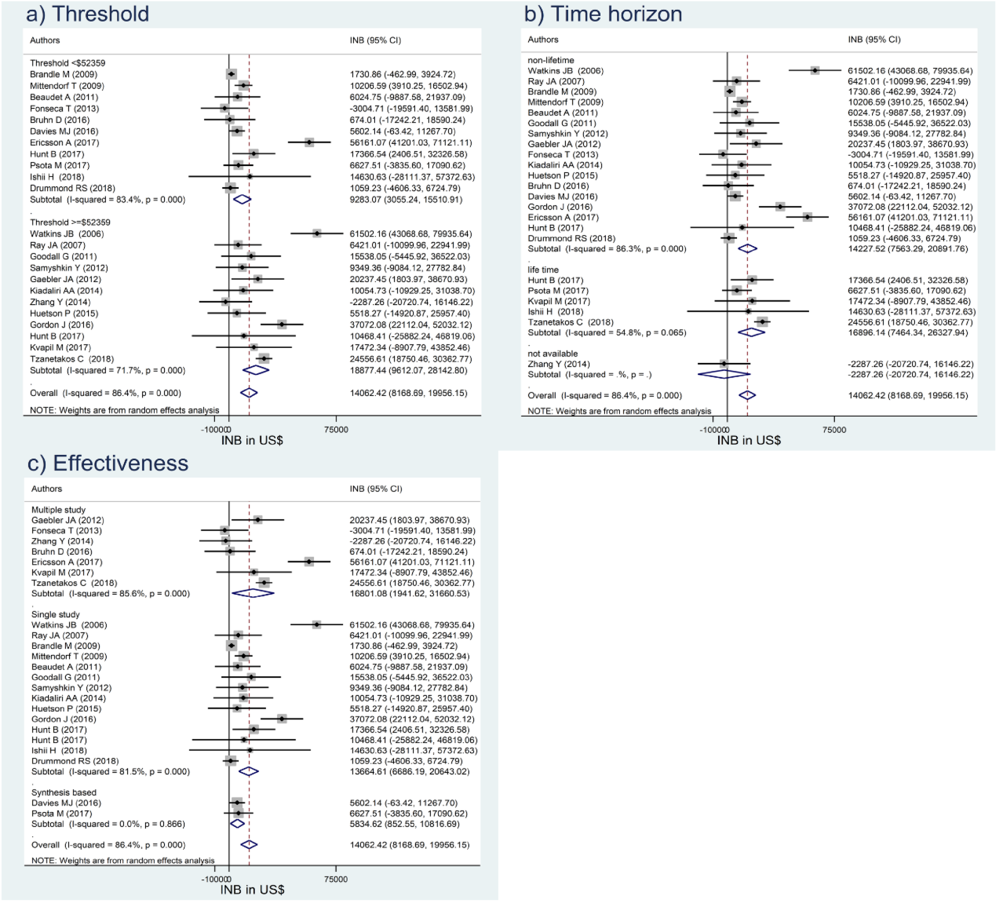

Suppl. Fig. 11 Sub-group analysis of pooling INB of GLP1 versus Insulin among HIC by a) high threshold b) low threshold c) effectiveness measure

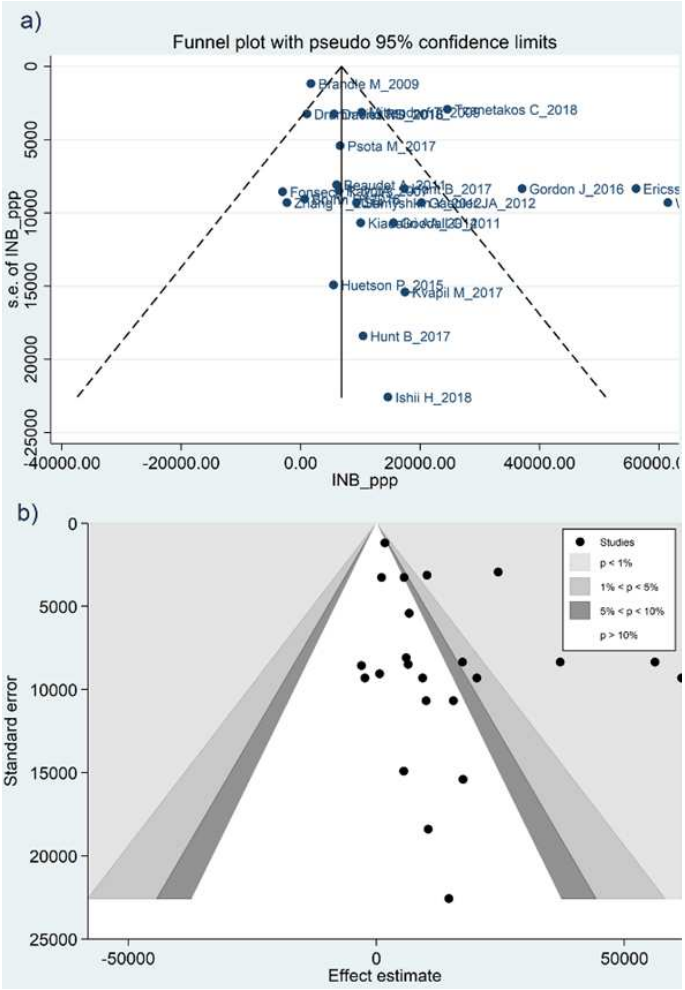

Suppl. Fig. 12 GLP1 versus Insulin among HIC a) funnel plot b) contour enhanced funnel plot

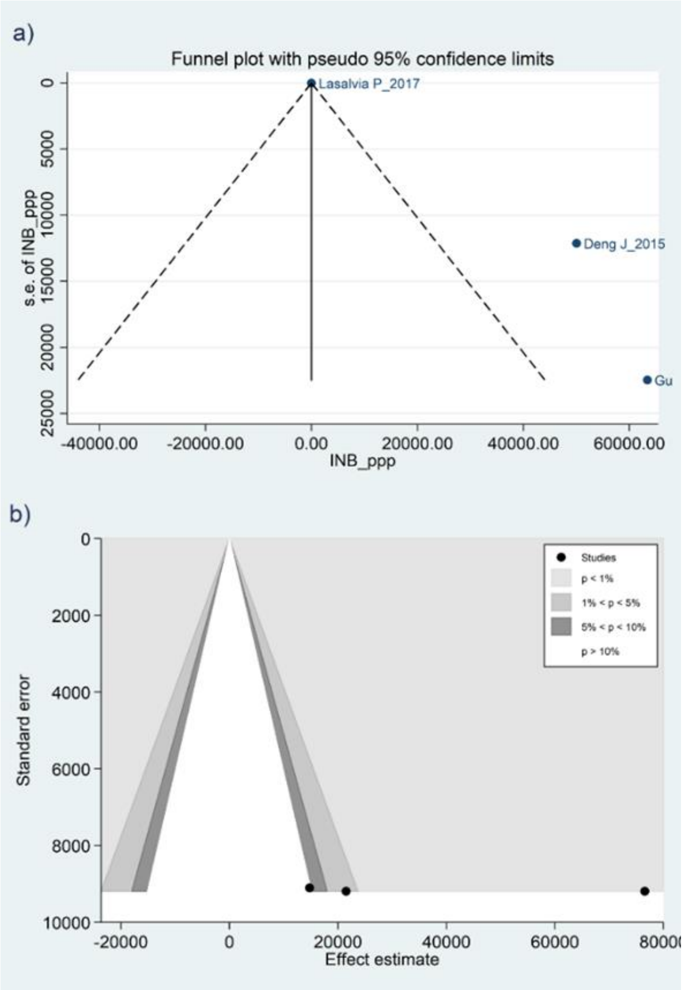

Suppl. Fig. 13 GLP1 versus Insulin in UMIC a) funnel plot b) contour enhanced funnel plot

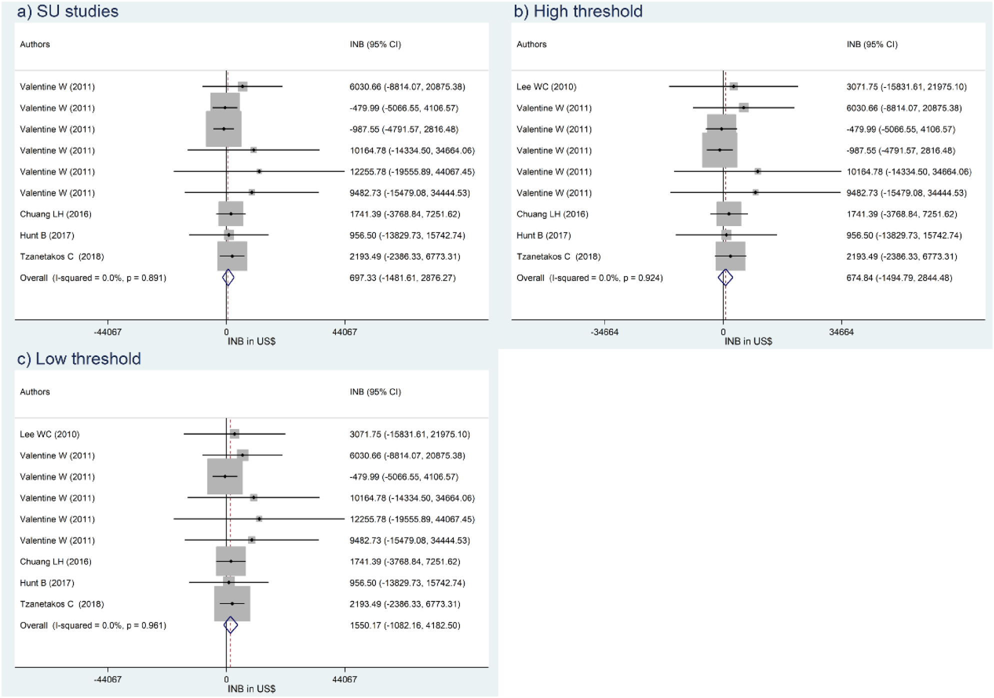

Suppl. Fig. 14 Sensitivity analysis of pooling INB of Liraglutide versus Exenatide by omitting studies with a) Exenatide & Sulfonylurea comparator b) high threshold c) low threshold

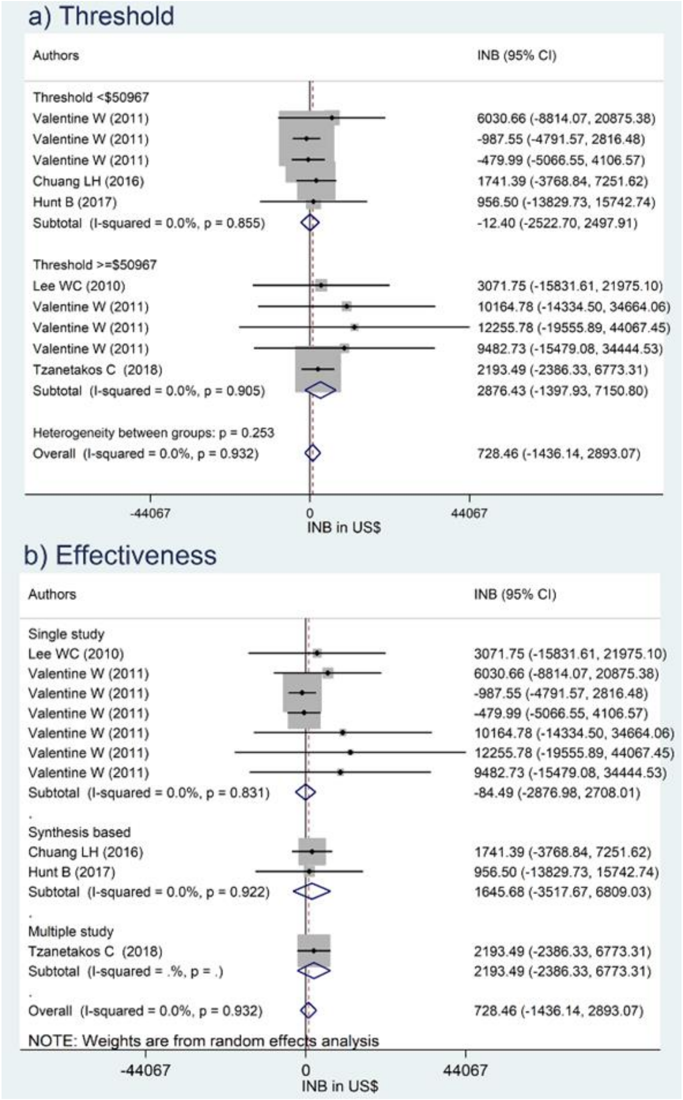

Suppl. Fig. 15 Subgroup analysis Liraglutide versus Exenatide by threshold

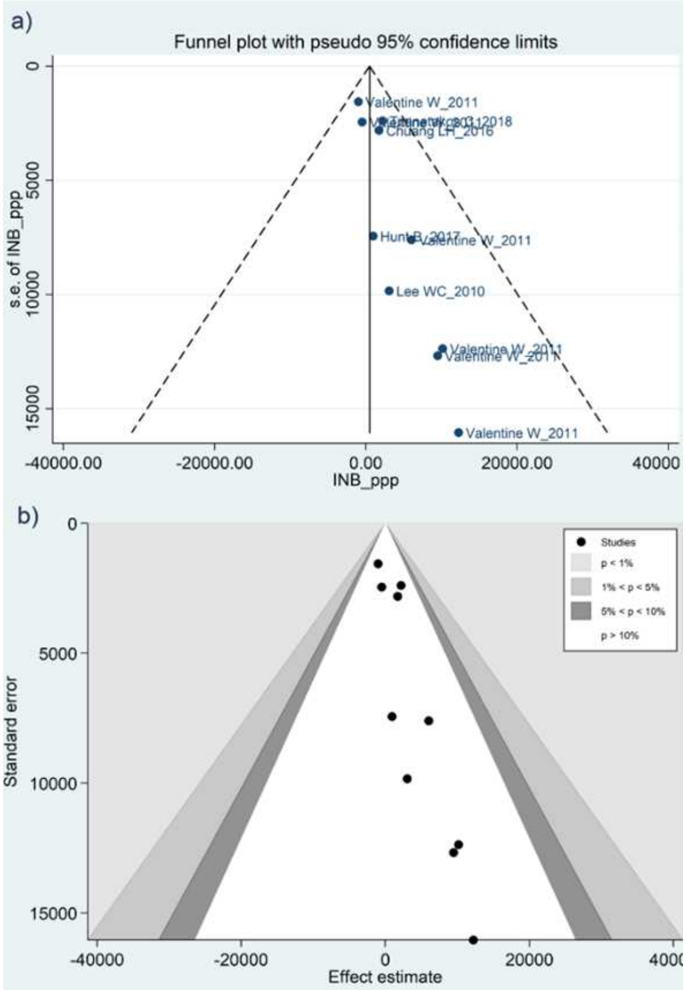

Suppl. Fig. 16 Liraglutide versus Exenatide a) funnel plot b) contour enhanced funnel plot

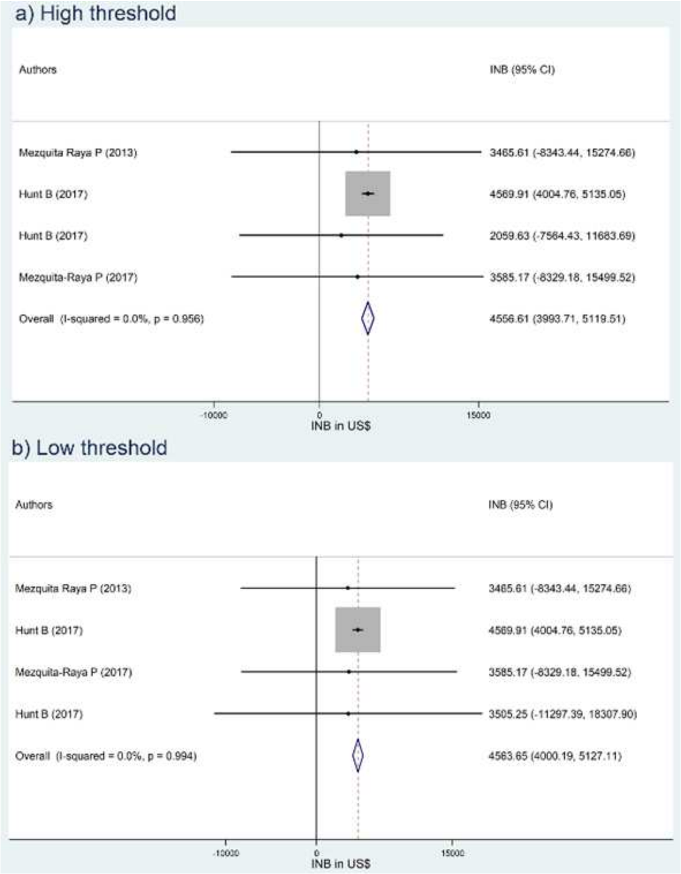

Suppl. Fig. 17 Sensitivity analysis of INB, Liraglutide versus Lixisenatide by omitting the studies with a) high threshold b) low threshold

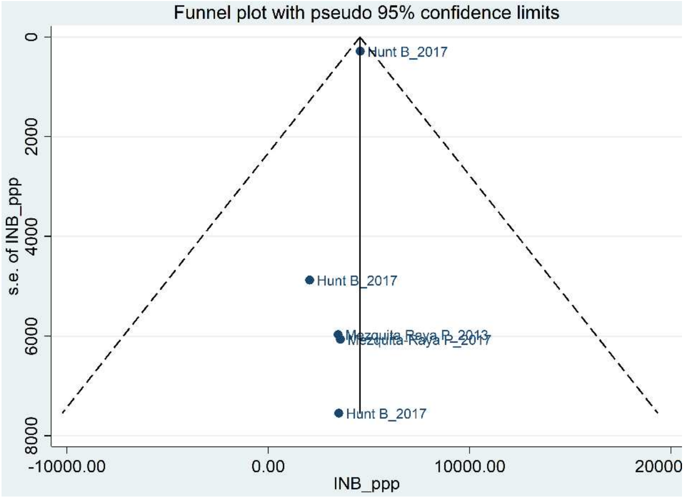

Suppl. Fig. 18 Liraglutide versus Lixisenatide funnel plot.

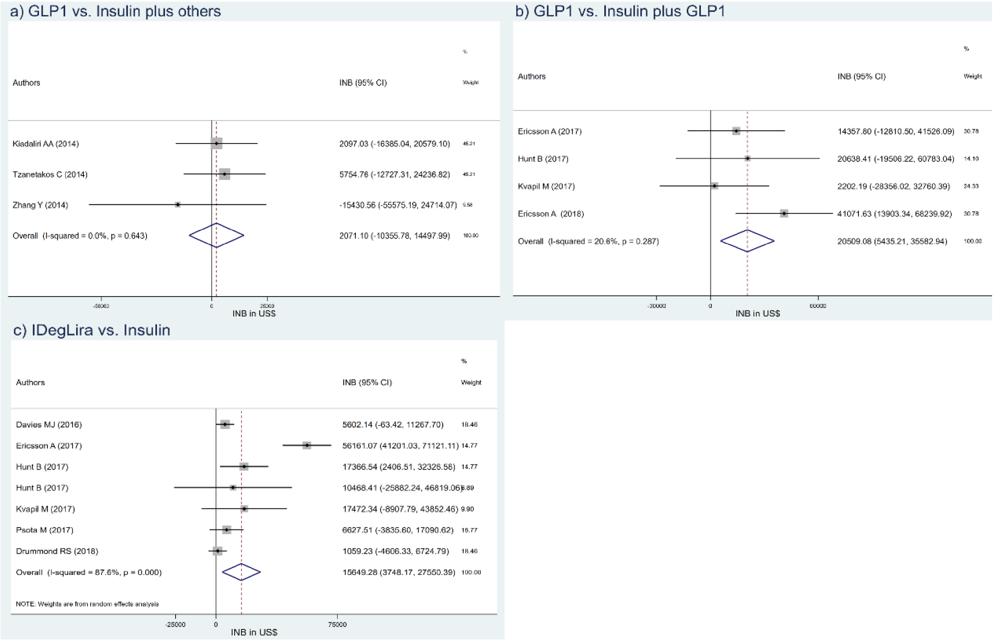

Suppl. Fig. 19 Pooling INB of a) GLP1 versus Insulin plus others b) GLP1 versus Insulin plus GLP1 c) IDegLira versus Insulin

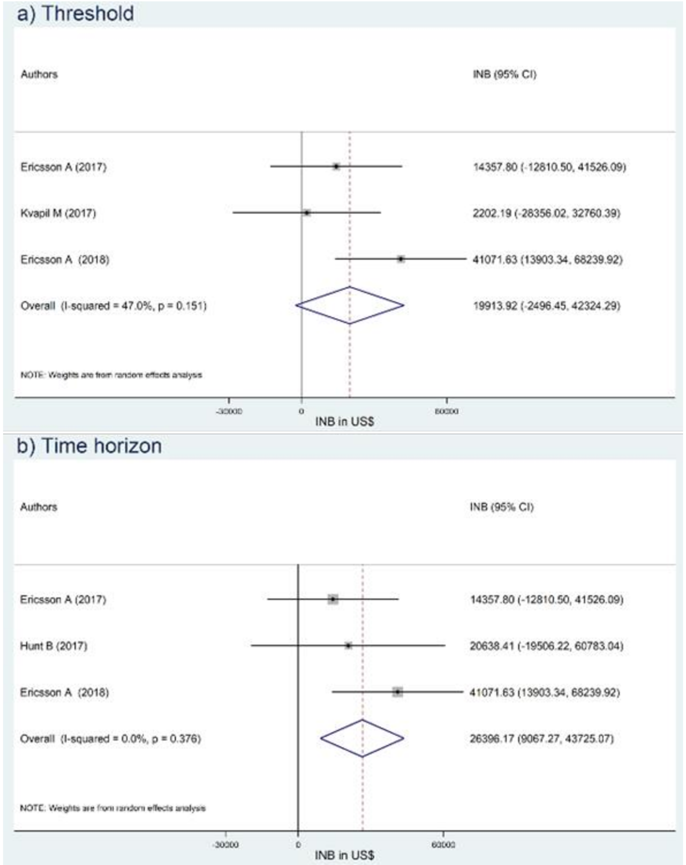

Suppl. Fig. 20 Sensitivity analysis of pooling INB of GLP1 versus Insulin plus GLP1 by omitting the studies with a) high threshold b) life-time horizon

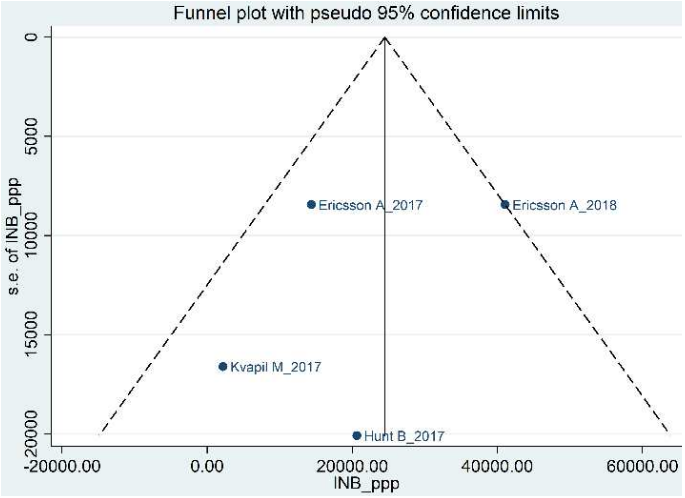

Suppl. Fig. 21 GLP1 versus Insulin plus GLP1 funnel plot

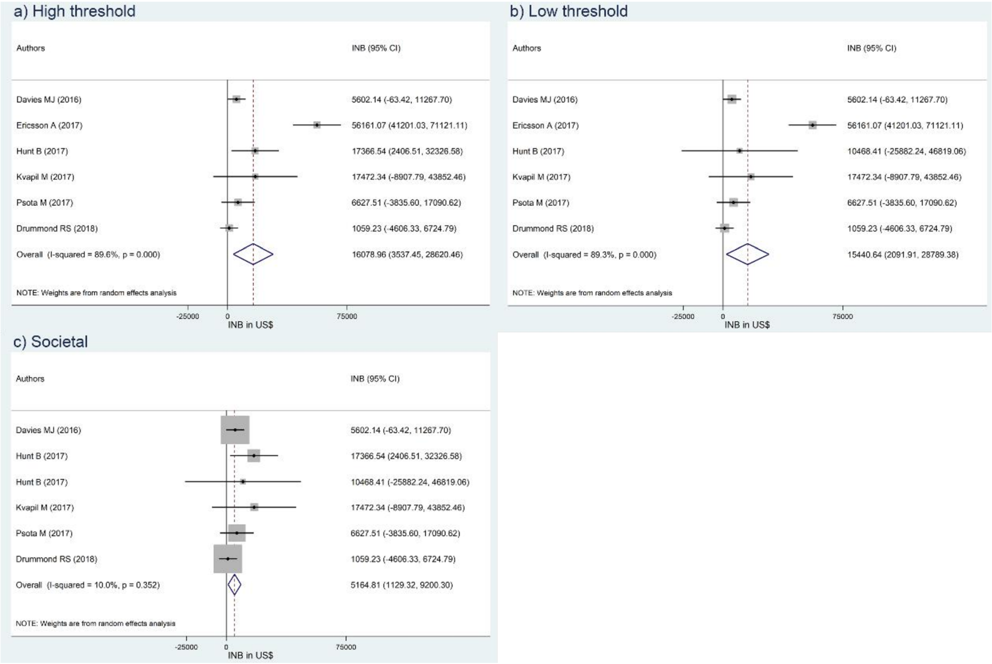

Suppl. Fig. 22 Sensitivity analysis of pooling INB of IDegLira versus Insulin by omitting the studies with a) high threshold b) low threshold c) societal perspective.

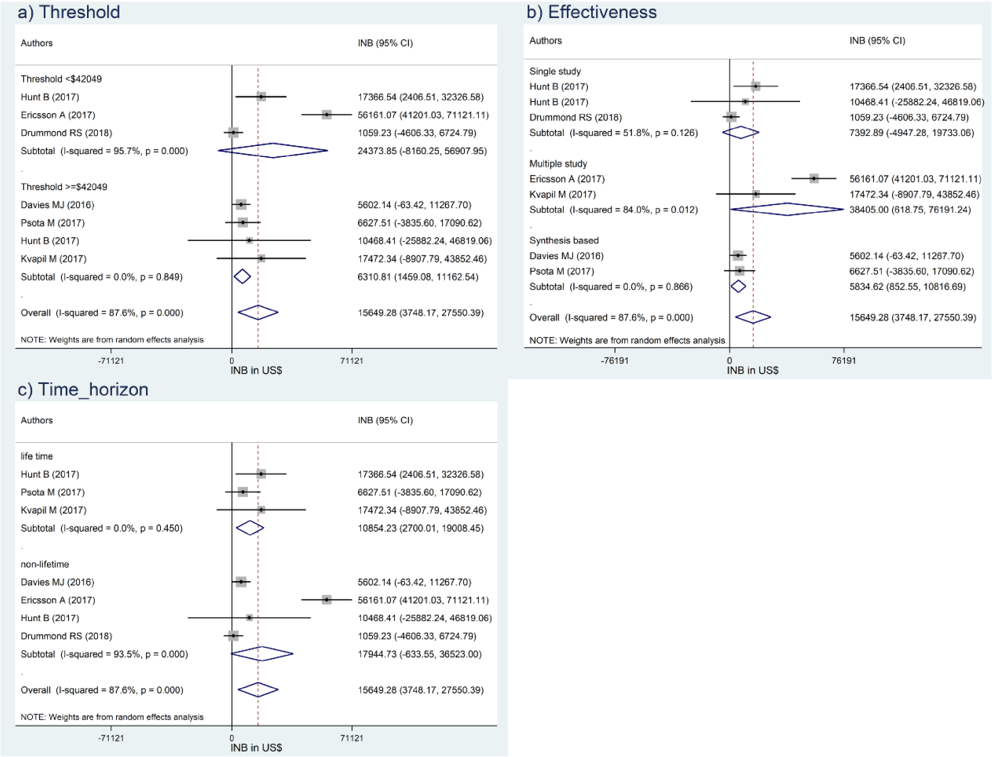

Suppl. Fig. 23 Subgroup analysis IDegLira versus Insulin by a) threshold b) effectiveness measure c) time horizon

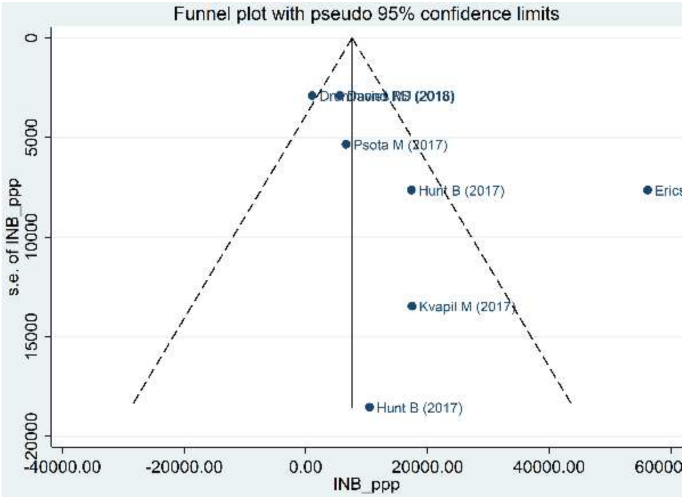

Suppl. Fig. 24 Funnel plot of IDegLira versus Insulin
